# Magnetic resonance elastography in nonlinear viscoelastic materials under load

**DOI:** 10.1007/s10237-018-1072-1

**Published:** 2018-08-27

**Authors:** Adela Capilnasiu, Myrianthi Hadjicharalambous, Daniel Fovargue, Dharmesh Patel, Ondrej Holub, Lynne Bilston, Hazel Screen, Ralph Sinkus, David Nordsletten

**Affiliations:** 1 Division of Biomedical Engineering and Imaging Sciences, King’s College London, London, UK; 2 Present Address: KIOS Research and Innovation Centre of Excellence, University of Cyprus, Nicosia, Cyprus; 3 Department of Biomedical Engineering and Cardiac Surgery, University of Michigan, Ann Arbor, USA; 4 Institute of Bioengineering, Queen Mary University of London, London, UK; 5 Prince of Wales Clinical School, University of New South Wales, Sydney, Australia; 6 Neuroscience Research Australia, Sydney, Australia; 7 Inserm U1148, LVTS, University Paris Diderot, University Paris 13, 75018 Paris, France

**Keywords:** Elastic waves, Tissue mechanics, Nonlinear mechanics, Magnetic resonance elastography, Biorheology

## Abstract

Characterisation of soft tissue mechanical properties is a topic of increasing interest in translational and clinical research. Magnetic resonance elastography (MRE) has been used in this context to assess the mechanical properties of tissues in vivo noninvasively. Typically, these analyses rely on linear viscoelastic wave equations to assess material properties from measured wave dynamics. However, deformations that occur in some tissues (e.g. liver during respiration, heart during the cardiac cycle, or external compression during a breast exam) can yield *loading bias*, complicating the interpretation of tissue stiffness from MRE measurements. In this paper, it is shown how combined knowledge of a material’s rheology and loading state can be used to eliminate loading bias and enable interpretation of *intrinsic* (unloaded) stiffness properties. Equations are derived utilising perturbation theory and Cauchy’s equations of motion to demonstrate the impact of loading state on periodic steady-state wave behaviour in nonlinear viscoelastic materials. These equations demonstrate how loading bias yields apparent material stiffening, softening and anisotropy. MRE sensitivity to deformation is demonstrated in an experimental phantom, showing a loading bias of up to twofold. From an unbiased stiffness of $4910.4 \pm 635.8$ Pa in unloaded state, the biased stiffness increases to 9767.5 $\pm \,$1949.9 Pa under a load of $\approx$ 34% uniaxial compression. Integrating knowledge of phantom loading and rheology into a novel MRE reconstruction, it is shown that it is possible to characterise intrinsic material characteristics, eliminating the loading bias from MRE data. The framework introduced and demonstrated in phantoms illustrates a pathway that can be translated and applied to MRE in complex deforming tissues. This would contribute to a better assessment of material properties in soft tissues employing elastography.

## Introduction

Diagnosis and therapy planning often depend on developing an accurate understanding of the state of a patient’s disease. Complex alterations occur in diseased tissue, depending on a range of factors such as changes to protein isoforms, alterations in protein density, extracellular matrix reorganisation, inflammation, etc. These modifications fundamentally impact tissue mechanical properties, triggering changes in stiffness, elasticity and viscosity (Yeh et al. 2002; Yin et al. 2009; Baiocchini et al. 2016). The ability to detect changes in tissue mechanical properties in vivo has the potential to significantly benefit clinical medicine, enabling a more thorough assessment of pathology as well as monitoring of treatment progression. This potential has lead to the development of a number of approaches for the noninvasive assessment of tissue mechanics.

Manual palpation is a classical approach of inspecting tissue for changes in elasticity in accessible organs (Weiss 2003; Nguyen et al. 2014). Advancements in medical imaging have led to the development of elastography—a modern quantitative technique for assessing the mechanical properties of internal organs. Elastography imaging is usually achieved using Ultrasound or Magnetic Resonance Imaging (MRI) (see Carey and Carey 2010 for a review), enabling the determination of tissue properties in vivo. Elastography imaging focuses on observing waves as they traverse through tissues, thus allowing quantification of strain response due to an excitation. In the case of magnetic resonance elastography (MRE), low-amplitude harmonic waves (10–1000, 30–80 Hz in vivo) are produced on the body and subsequently imaged in three-dimensions (Manduca et al. 2001; Fovargue et al. 2018b). Harmonic wave motion is then related to local tissue properties, in most cases, through linear viscoelastic wave equations (Glaser et al. 2012). MRE has been successfully employed for a range of tissues—including brain (Klatt et al. 2007; Green et al. 2008; Schregel et al. 2012), liver (Huwart et al. 2006, 2007, 2008; Klatt et al. 2007) and breast (Sinkus et al. 2000, 2005a, 2007; Houten et al. 2003)—showing contrast between diseased and healthy tissue as well as providing resolution of apparent tissue anisotropy (Qin et al. 2013). More recently, it has also been applied to heart (Elgeti et al. 2008; Kolipaka et al. 2009; Robert et al. 2009; Kolipaka et al. 2010; Couade et al. 2011; Kolipaka et al. 2012; Elgeti and Sack 2014), skeletal muscles (Green et al. 2012, 2013), and other soft tissues, reviewed in Manduca et al. (2001), Mariappan et al. (2010) and Fovargue et al. (2018b).

While interpreting stiffness measures in organs, such as the liver, has shown diagnostic value, a challenge yet to be addressed is the impact of macroscale deformations on wave propagation behaviour. Broadly, by looking at the 1D wave equation $\partial ^2 u / \partial t^2 = c^2 \partial ^2 u / \partial x^2$, term $c = \sqrt{T/\rho }$ depends on tension *T* and linear density $\rho$ and is scaling the spatial second derivative of displacement *u*. The resultant force term on the right hand side balances the acceleration term on the left hand side, leading to the phase velocity increasing with higher tension. Elastic, hyperelastic and viscoelastic nonlinear materials would exhibit this effect. This is relevant in the context of tissues that undergo natural deformations (e.g: the heart muscle during the cardiac cycle (Asner et al. 2017), the liver during respiration (Kang et al. 2012), etc.) and tissues that undergo imposed deformations (e.g. breast during MRE scans Sinkus et al. 2005a, 2007). As many tissues are known to exhibit significant nonlinearity (Nash and Hunter 2000; Taber 2004; Liu et al. 2006; Holzapfel and Ogden 2009; Gao et al. 2010;  Nordsletten et al. 2011b; Goenezen et al. 2012) under physiological conditions, both natural and imposed deformation can result in *loading bias*—an apparent stiffness that is a conglomerate measure of the intrinsic material properties and current kinematic state. For instance, the nonlinearity arising from compressive strain was quantified in ex vivo bovine liver samples using MRE measurements (Clarke et al. 2011). Hence, mixtures of patient-specific kinematics and intrinsic tissue properties are non-trivial and presumably result in a reduction in clinical specificity.

In this paper, the harmonic perturbation of Cauchy’s equations of motion for a general incompressible nonlinear viscoelastic material is examined, in order to understand the links between intrinsic properties and kinematics in MRE. From this analysis, a set of governing equations are derived, illustrating the general impact of large deformation on the motion of harmonic waves. Importantly, these equations enable the elimination of loading bias and therefore retrieval of intrinsic properties when large-scale deformation and constitutive behaviour are known. Theoretical test cases are presented, showing the ability of deformation to yield apparent increase, decrease and anisotropy in materials. In order to demonstrate the applicability of these perturbed equations, experiments were performed in polyvinyl alcohol (PVA) phantoms. Rheological tests were performed to characterise material behaviour, and loaded phantoms were imaged using MRE. Results show the capacity of these equations to correct for the complex dynamics of wave motion in deformed materials. To the authors’ knowledge, this is the first cross-validation study linking mechanical characterisation of a material in rheology with wave motion through the deformed material in MRE. The developments presented provide a key step towards improving the estimation of material properties in soft tissues, using elastography.

As a starting point, Sect. 2.1 presents perturbation theory employed within Cauchy’s equations of motion. The perturbed equations are analysed in the context of a general viscoelastic material under load, further leading to the derivation of apparent stiffness moduli influenced by large deformations and material constitutive law. For exemplification purposes, an idealised case of harmonic wave motion traversing a uniformly compressed Neo-Hookean material is presented. Experiments are employed to test the theoretical ground developed. In Sect. 2.2, PVA phantoms are tested in a rheological setup, to determine the material governing law. In Sect. 2.3, MRE data are acquired in PVA phantoms at different uniaxial compression levels. The knowledge on rheological behaviour and deformation is integrated into MRE data analysis, in order to undo the loading bias. The experimental results are presented in Sect. 3. Particularly, the estimation of the intrinsic stiffness of PVA is presented in Sect. 3.3. The theoretical framework presented in this study could be extended to in vivo MRE data analysis.

## Materials and methods

### Harmonic wave motion in deformed nonlinear viscoelastic materials

MRE relies on the analysis of small amplitude harmonic waves traversing through materials. As materials undergo deformation, wave behaviour is impacted (Thurston and Brugger 1964). One option to understand the relationship between large-scale deformation and small-scale wave behaviour is perturbation analysis. After covering notation 2.1.1, this section reviews perturbation results for Cauchy’s equations (Sect. 2.1.2) considering the particular case of a nonlinear viscoelastic material. Example cases are then highlighted (Sect. 2.1.3), illustrating the impact of load on the apparent stiffness characteristics stemming from MRE waves.

#### Kinematics, harmonic wave motion and notation

Here, we briefly review the basic kinematics in large deformation mechanics in order to later describe the macroscale deformation of phantom and tissue material subjected to MRE. For more complete dispositions, see for example (Malvern 1969; Wang and Truesdell 1973; Graff 1991; Holzapfel 2000; Bonet and Wood 2008). The motion of a solid body, denoted by the region ${\varOmega }_0\subset {\mathbb {R}}^d$, is characterised by the displacement field ${\varvec{U}}: {\varOmega }_0\times [0, T] \rightarrow {\mathbb {R}}^d$. In this case, any reference point $\varvec{X} \in {\varOmega }_0$ may be deformed to its physical position by the diffeomorphic Lagrangian mapping ${\mathcal {L}}: {\varOmega }_0\times [0, T] \rightarrow {\mathbb {R}}^d$ so that its position at a time $t \in [0,T]$ is given by

$$\begin{aligned} \varvec{x} = {\mathcal {L}}(\varvec{X},t) = \varvec{U}(\varvec{X},t) + \varvec{X}, \quad \forall \varvec{X} \in {\varOmega }_0. \end{aligned}$$

Due to the Lagrangian mapping, note that any *m*-dimensional function ${\varvec{v}} : {\varOmega }_0\times [0, T] \rightarrow {\mathbb {R}}^m$ can be written as a function of the deformed domain ${\varOmega }(t)$, e.g. $\varvec{\widetilde{v}} : {\varOmega }\rightarrow {\mathbb {R}}^m$, with the understanding that $\varvec{\widetilde{v}}({\varvec{x}}, t) = {\varvec{v}}({\mathcal {L}}^{-1}({\varvec{x}},t),t)$. For ease of notation, the $\widetilde{\cdot }$ is dropped where there is clear distinction.

The stresses resulting from material deformation can be separated into deviatoric and hydrostatic components. For the latter, the effect is quantified using the hydrostatic pressure *P*, which ensures some constraint on the volume or pressure-volume relation. The deviatoric components are, in turn, related to shape changes (or strains and strain history).

Important quantities for mechanics are the deformation gradient tensor $\varvec{F}$ and its determinant *J*:

$$\begin{aligned} \begin{aligned} \varvec{F}= \nabla _0 {\varvec{U}}+ \varvec{I} , \,\,\, J(\varvec{F}) = \det \varvec{F}. \end{aligned} \end{aligned}$$

Here, $\nabla _0$ refers to the gradient operator taken with respect to coordinates of ${\varOmega }_0$, so that $(\nabla _0)_k = \partial / \partial X_k$. For later use, $J = J(\varvec{F})$ is implied.

When relating kinematics and kinetics, the right or left Cauchy Green strains

$$\begin{aligned} \varvec{C}= \varvec{F}^T \varvec{F}, \quad \varvec{B}= \varvec{F}\varvec{F}^T \end{aligned}$$

are often considered. These may also be defined in terms of their isochoric variants (Ogden 1997; Bonet and Wood 2008) as

$$\begin{aligned} {\hat{\varvec{F}}} = J^{-1/d} \varvec{F}, \quad {\hat{\varvec{C}}} = {\hat{\varvec{F}}}^T {\hat{\varvec{F}}}, \quad {\hat{\varvec{B}}} = {\hat{\varvec{F}}}{\hat{\varvec{F}}}^T, \end{aligned}$$

which are invariant to changes in volumetric properties of the material, i.e. ${\hat{\varvec{C}}}(a \varvec{F}) = {\hat{\varvec{C}}}(\varvec{F})$ for a real positive scalar $a \in {\mathbb {R}}^+$ (Holzapfel 2000). A set of invariants are associated with these quantities. Here, the first and second invariants of any second-order tensor ${\varvec{A}} \in {\mathbb {R}}^{m \times m}$ are defined as in Bonet and Wood (2008)

$$\begin{aligned} I_{{\varvec{A}}} = {\varvec{A}}:{\varvec{I}}, \quad II_{{\varvec{A}}} = {\varvec{A}}:{\varvec{A}}. \end{aligned}$$

For later clarity, the double contraction “:” is used as defined in Bonet and Wood (2008), e.g. the contraction of second-order square tensors ${\varvec{A}} ,{\varvec{B}} \in {\mathbb {R}}^{m \times m}$ becomes a scalar^1^

$$\begin{aligned} {\varvec{A}} : {\varvec{B}} = A_{ij}B_{ij}, \end{aligned}$$

and the contraction of a fourth-order tensor $\varvec{\mathcal {A}} \in {\mathbb {R}}^{m \times m \times m \times m}$ and a second-order tensor ${\varvec{B}}$ yields a second-order tensor in ${\mathbb {R}}^{m \times m}$^2^

$$\begin{aligned} ({\varvec{\mathcal {A}}} : {\varvec{B}})_{ij} = A_{ijkl}B_{kl}. \end{aligned}$$

For further use throughout the paper, the multiplication of second- and fourth-order tensors ${\varvec{A}} {\varvec{\mathcal {A}}}$ or ${\varvec{\mathcal {A}}} {\varvec{B}}$ is defined to take place over the last index of the first quantity and first index of the second quantity, i.e.

$$\begin{aligned} ({\varvec{A}} {\varvec{\mathcal {A}}} )_{ijkl} = A_{is} {\mathcal {A}}_{sjkl}, \\ ( {\varvec{\mathcal {A}}} {\varvec{B}} )_{ijkl} = {\mathcal {A}}_{ijks} B_{sl}. \end{aligned}$$

Finally, the derivative of a second-order tensor with respect to another is defined as

$$\begin{aligned} (\nabla _{{\varvec{B}}} {\varvec{A}})_{ijkl} = \frac{\partial A_{ij}}{\partial B_{kl}}. \end{aligned}$$

MRE relies on the propagation of shear waves through the body, assumed to reach a harmonic steady state. Being wave motion, these deformations are assumed to be linear and small. The large deformation ${\varvec{U}}$ is perturbed by these small wave deformations

1 $$\begin{aligned} \varvec{u}^{\varepsilon }: {\varOmega }_0\times [0,T] \rightarrow {\mathbb {R}}^d, \end{aligned}$$

where the scale of the perturbations is much smaller than the dominant length scale of the body or the large displacement it undergoes. When the perturbations are periodic-in-time and have reached a steady-state, the deformations and pressures can be written as

2 $$\begin{aligned} \varvec{u}^{\varepsilon }= \text {Re} \{ \varvec{u}_{{c}}e^{i \omega t} \} , \qquad p^{\varepsilon }= \text {Re} \{ p_{{c}}e^{i \omega t} \} , \end{aligned}$$

where $$ \varvec{u}_{{c}}= \varvec{u}_{r}+ i \varvec{u}_{i}$$ ($$ \varvec{u}_{r}, \varvec{u}_{i}: {\varOmega }_0\rightarrow {\mathbb {R}}^d $$) and $$ p_{{c}}= p_{r}+ i p_{i}$$ ($$ p_{r}, p_{i}: {\varOmega }_0\rightarrow {\mathbb {R}} $$) give the complex periodic steady-state behaviour with real and imaginary parts given by subscripts *r* and *i*, respectively.

#### Perturbation of Cauchy’s equations of motion

For an incompressible body, at any time $t \in [0,T]$, motion of ${\varOmega }_0$ satisfies Cauchy’s first law (conservation of momentum) and conservation of mass shown in Eqs. 3a and 3b, respectively (see Malvern 1969; Ogden 1997; Holzapfel 2000; Bonet and Wood 2008):

3a $$\begin{aligned}&\rho J \partial _{tt} {\varvec{U}}- \nabla _0 \cdot (\varvec{F}{\varvec{S}}) = \varvec{0} \text { on } {\varOmega }_0, \end{aligned}$$

3b $$\begin{aligned}&J - 1 = 0 \text { on } {\varOmega }_0. \end{aligned}$$

Here, $\rho$ is the material density, $\partial _{tt}$ is the second time derivative, and ${\varvec{S}}_e = {\varvec{S}} (P, \varvec{C})$ is the second Piola Kirchhoff (PK2) stress tensor depending on the hydrostatic pressure, *P*, and the right Cauchy Green tensor, $\varvec{C}$.

The PK2 stress can be characterised as elastic, hyperelastic, viscoelastic, etc., depending on the constitutive relation that describes the material. A hyperelastic material, for example, can be written as a function of $\varvec{C}$ (or other strain metrics) and space ($\varvec{X}$). In contrast, a viscoelastic material additionally depends on time (*t*). We recall briefly that, for hyperelastic materials—such as those commonly applied in biomechanics—${\varvec{S}}_e$ is given as the derivative of a governing hyperelastic strain energy function, $W = W_e(\varvec{C})$ (Bonet and Wood 2008), with respect to the right Cauchy Green tensor $\varvec{C}$, i.e.

4 $$\begin{aligned} {\varvec{S}}_e = 2\nabla _{\varvec{C}}W_e, \quad S_{e,ij} = 2 \frac{\partial W_e}{\partial C_{ij}}. \end{aligned}$$

This model is extensively applied in nonlinear biomechanics—for example in simulations of the heart (Wang et al. 2009; Nordsletten et al. 2011a; McCormick et al. 2013, 2014; Hadjicharalambous et al. 2014), breast (Rajagopal et al. 2010; Reynolds et al. 2011; Gamage et al. 2011), arterial wall (Holzapfel 2000; Gasser et al. 2006; Hariton et al. 2007), etc.-where specific material response is usually defined through biorheological experiments. The simplest material models are purely elastic, based on spring rheological elements. Complementary, pure viscosity is based solely on dashpot elements. Biological tissues usually exhibit viscoelasticity; hence, a suitable tissue model should consider a combination of these springs and dashpots. For instance, the Maxwell model is formed by considering a spring and dashpot connected in series, whereas the Kelvin–Voigt model is formed by considering a spring and dashpot connected in parallel. These types of models have been analysed in literature (Liu and Bilston 2000; Bilston et al. 2001; Banks et al. 2011), but have shortcomings in predicting creep (Maxwell model) or stress relaxation (Kelvin–Voigt model) (Liu et al. 2006; Banks et al. 2011). While the Maxwell constitutive equation was found to be better suited for modelling fluids (Houten et al. 2000; Sinkus et al. 2005a), the Kelvin–Voigt constitutive equation was employed for purposes similar to ours, modelling soft tissues subjected to elastography testing (Sinkus et al. 2005a; Huwart et al. 2006; Sinkus et al. 2007; Huwart et al. 2008) (albeit not considering the effects of large deformations on the wave behaviour). However, the Kelvin–Voigt model is not able to accurately capture the power-law dependence on frequency that is usually observed in tissues (Chui et al. 2004; Sinkus et al. 2007; Nicolle et al. 2010; Nicolle 2015). Thus, an adaptation of this model is commonly used, which replaces the dashpot with a springpot. This form was observed to describe tissue better (Kiss et al. 2004).

To capture the viscoelastic behaviour exhibited generally by tissues and polymers, we proceed by assuming that the PK2 stress can be decomposed into an additive sum of elastic, viscoelastic and hydrostatic components, i.e.

5 $$\begin{aligned} {\varvec{S}} = {\varvec{S}}_{e} + {\varvec{S}}_p + D_t^\alpha {\varvec{S}}_{v}. \end{aligned}$$

The elastic part ${\varvec{S}}_{e}$ is defined through a hyperelastic strain energy function $W_e(\varvec{C})$ as in Eq. 4, the hydrostatic part as ${\varvec{S}}_{p} = J P {\varvec{C}}^{-1}$, and the viscoelastic part is defined using the Caputo formulation (Caputo 1967) of the fractional-order derivative:

$$\begin{aligned} D_t^\alpha {\varvec{S}}_{v} = \frac{1}{{\varGamma }(1-\alpha )} \int _0^t \frac{1}{(t-z)^\alpha }\partial _t{\varvec{S}}_{v}(z) \text { d} z. \end{aligned}$$

Here, ${\varvec{S}}_{v}$ depends on a viscoelastic strain energy function such as ${\varvec{S}}_{v} = 2\nabla _{\varvec{C}}W_v$. Considering the coefficient of derivation $\alpha$ to be 0, the viscoelastic stress ${\varvec{S}}_v$ acts as a hyperelastic term, while $\alpha =1$ indicates the first-order derivative $\partial _t{\varvec{S}}_v$. Intermediate values of $\alpha$ would lead to non-trivial transitional results between ${\varvec{S}}_v$ and $\partial _t{\varvec{S}}_v$. Therefore, this formulation of the viscoelastic term leads to a fractional nonlinear Kelvin–Voigt type of model and the PK2 tensor for our viscoelastic material becomes

6a $$\begin{aligned} {\varvec{S}}= & {} 2\nabla _{\varvec{C}}W_e + 2 D_t^\alpha (\nabla _{\varvec{C}}W_v) + JP\varvec{C}^{-1}, \end{aligned}$$

6b $$\begin{aligned} S_{ij}= & {} 2 \frac{\partial W_e}{\partial C_{ij}} + 2 D_t^\alpha \bigg ( \frac{\partial W_v}{\partial C_{ij}} \bigg ) + JP C^{-1}_{ij}. \end{aligned}$$

In order to investigate the effect of deformation on wave behaviour, we consider the case where high-frequency/low-amplitude harmonic waves (see Eq. 1) are imposed onto our body that satisfies the set of Eq. 3. For our purposes, we assume that the introduction of these micro-deformations $\varvec{u}^{\varepsilon }$ does not disrupt the natural state of macro-deformation $\varvec{U}$, such that the total observed state $({\varvec{U}}^\varepsilon , P^\varepsilon )$ can be characterised by

7 $$\begin{aligned} {\varvec{U}}^\varepsilon (\varvec{X},t)= & {} {\varvec{U}}(\varvec{X},t) + \varvec{u}^{\varepsilon }(\varvec{X},t) , \nonumber \\ P^\varepsilon (\varvec{X},t)= & {} P(\varvec{X},t) + p^{\varepsilon }(\varvec{X},t) , \end{aligned}$$

with $({\varvec{U}}, P)$ satisfying the original Eq. 3 (without perturbation conditions) and with $({\varvec{U}}^\varepsilon , P^\varepsilon )$ satisfying a perturbed version of Eq. 3, i.e.

8a $$\begin{aligned}&\rho J^\varepsilon \partial _{tt} {\varvec{U}}^\varepsilon - \nabla _0 \cdot (\varvec{F}^\varepsilon {\varvec{S}}^\varepsilon ) = \varvec{0} \quad \text { on } \quad {\varOmega }_0, \end{aligned}$$

8b $$\begin{aligned}&J^\varepsilon - 1 = 0 \quad \text { on } \quad {\varOmega }_0, \end{aligned}$$

where superscript $\varepsilon$ indicates quantities dependent on the perturbed state variables.

A simplification of the set of Eq. 8 can be achieved by expanding about the state variable ${\varvec{U}}$ and then linearising with respect to the small perturbations $\varvec{u}^{\varepsilon }$, $p^{\varepsilon }$ (Thurston and Brugger 1964). For completeness, details about the expansion and linearisation processes can be found in Appendix 1. Considering the case of harmonic waves, a set of periodic nonlinear viscoelastic wave equations can be derived in the reference domain ${\varOmega }_0$. By recasting the reference frame gradient and divergence operators into their physical domain counterparts, the set of Eq. 8 can be transformed into the physical domain as:

9a $$\begin{aligned}&-\rho \omega ^2 \varvec{u}_{{c}}- \nabla \cdot [ (\varvec{\mathcal {G}} ^\prime + i \varvec{\mathcal {G}} ^{\prime \prime } ): \nabla \varvec{u}_{{c}}+ p_{{c}}\varvec{I}] = \varvec{0}, \end{aligned}$$

9b $$\begin{aligned}&\nabla \cdot \varvec{u}_{{c}}= 0 , \end{aligned}$$

where $\nabla = \partial / \partial x$ on ${\varOmega }$. Here, $\varvec{\mathcal {G}} ^\prime$ and $\varvec{\mathcal {G}} ^{\prime \prime }$ are the real and imaginary stiffness moduli influencing the apparent wave dynamics of $\varvec{u}_{{c}}$ and taking the form of fourth-order tensors:

10a $$\begin{aligned} {\mathcal {G}}^{\prime }_{ijml}= & {} \frac{1}{J} \Big [{\varvec{F}} \nabla _{\varvec{F}}({\varvec{S}}_e + \omega ^\alpha \cos \left( \frac{\pi \alpha }{2} \right) {\varvec{S}}_v \nonumber \\&+\, {\varvec{S}}_p ) + \varvec{{\mathcal {I}}} {\varvec{S}} \Big ]_{ismn} F_{ln} F_{js}, \end{aligned}$$

10b $$\begin{aligned} {\mathcal {G}}^{\prime \prime }_{ijml}= & {} \frac{\omega ^\alpha }{J} \sin \left( \frac{\pi \alpha }{2} \right) \big ( {\varvec{F}} \nabla _{\varvec{F}}{\varvec{S}}_v \big )_{ismn} F_{ln} F_{js} , \end{aligned}$$

where ${\varvec{F}}$, ${\varvec{S}}_e$ and ${\varvec{S}}_v$ depend on the unperturbed macro-deformation ${\varvec{U}}$ and hydrostatic pressure and *P*. In the absence of deformation, under the assumption of an isotropic material defined by a standard law (e.g. a Neo-Hookean model for ${\varvec{S}}_e$ and ${\varvec{S}}_v$), these stiffness moduli are simply given by $\varvec{\mathcal {G}} ^\prime + i \varvec{\mathcal {G}} ^{\prime \prime } = G^* \varvec{{\mathcal {I}}}$, where $G^*$ is the true complex shear modulus characterising the material and ${\mathcal {I}}_{ijkl} = \delta _{ik} \delta _{jl}$. The wave dynamics become symmetric, since $\varvec{{\mathcal {I}}} : \nabla \varvec{u}_{{c}}= \nabla \varvec{u}_{{c}}+\nabla \varvec{u}_{{c}}^T$. However, under deformation, the components of $\nabla \varvec{u}_{{c}}$ are scaled in a non-trivial way. By understanding the form of the scaling influencing the $\varvec{\mathcal {G}} ^\prime$ and $\varvec{\mathcal {G}} ^{\prime \prime }$ moduli, the bias introduced by deformation on the wave behaviour can be predicted. Furthermore, the deformation can be undone, that is, the moduli that reflect the undeformed state can be recovered.

#### Loading bias of planar shear waves in pure compression 

For an intuitive illustration, let us consider the case of a simple isotropic Neo-Hookean material described by the strain energy function

$$\begin{aligned} W_e(P, \varvec{F}) = \frac{\mu _e}{2} ( I_{{\hat{\varvec{C}}}} - 3 ), \end{aligned}$$

where $\mu _e$ is the elastic material parameter. The elastic stress is derived to be

$$\begin{aligned} {\varvec{S}}_e = \dfrac{\mu _e}{J^{2/3}} \left( {\varvec{I}} - \dfrac{I_{\varvec{C}}}{3}\varvec{C}^{-1} \right) . \end{aligned}$$

The viscous term is assumed to be zero, i.e. ${\varvec{S}}_v = {\varvec{0}}$. Under these assumptions, the real modulus $\varvec{\mathcal {G}} ^ \prime$ becomes

11 $$\begin{aligned} {\mathcal {G}}^\prime _{ijml}= & {} \frac{1}{J} \Bigg [ \mu _e {\hat{B}}_{lj} \delta _{im} - \frac{2\mu _e}{3} ({\hat{B}}_{ij} \delta _{ml} + {\hat{B}}_{ml} \delta _{ij}) \nonumber \\&+\, \delta _{ml} \delta _{ij} \left( \frac{2 \mu _e I_{{\hat{\varvec{B}}}}}{9} + PJ \right) \nonumber \\&+\, \delta _{mj} \delta _{il} \left( \frac{\mu _e I_{{\hat{\varvec{B}}}}}{3} - PJ \right) \Bigg ]. \end{aligned}$$

**Fig. 1:**
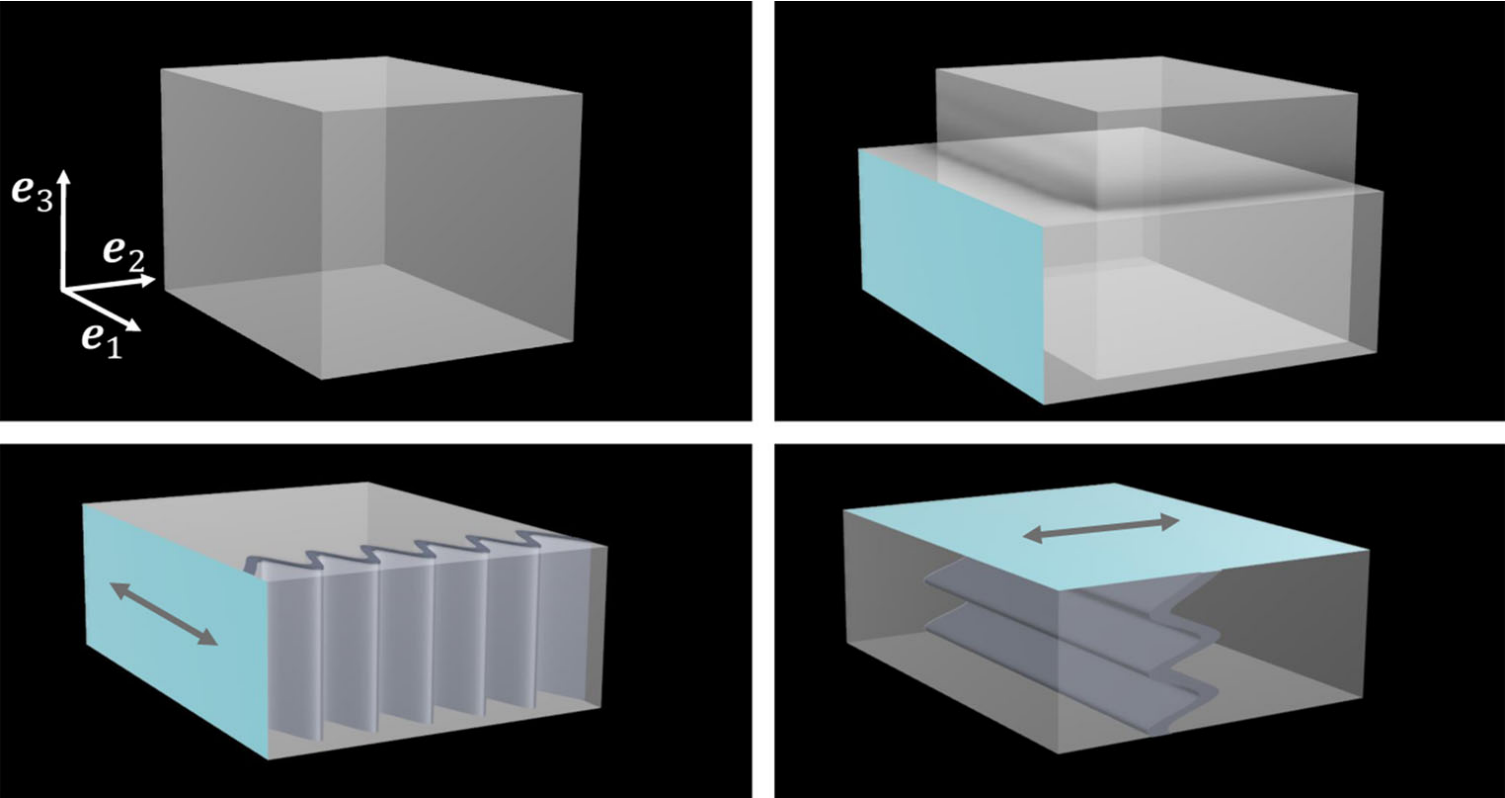
Planar waves through a loaded phantom; (Top left) Phantom in undeformed state. (Top right) Phantom in compressed state, compared against the undeformed state. (Bottom left) Planar wave created by moving the front face of the phantom (in blue) along the ${\varvec{e}}_1$ direction. (Bottom right) Planar wave created by moving the top face of the phantom (in blue) along the ${\varvec{e}}_2$ direction

To better understand the changes introduced by large deformations ${\varvec{U}}$ on the wave dynamics, we analyse the case of plane waves through a material under pure compression. Thus, considering a cuboid that is compressed along the height (${\varvec{e}}_3$ direction), imposing incompressibility, the deformation ${\varvec{U}}$ is described by

$$\begin{aligned} {\varvec{U}} = \big [ (1/\sqrt{\lambda }-1) X_1, \, (1/\sqrt{\lambda }-1) X_2, \, (\lambda -1) X_3 \big ]^T, \end{aligned}$$

where $\lambda$ is the ratio of the final height over the initial height (i.e. $\lambda <1$). In this case, the hydrostatic pressure is a constant given by $P= \mu _e (1/\lambda - \lambda ^2)/3$ and the kinematic tensors describing this deformation are

12 $$\begin{aligned} {\varvec{F}}= & {} \left( \begin{array}{ccc} {\frac{1}{\sqrt{\lambda }}}&{} 0 &{} 0 \\ 0 &{} {\frac{1}{\sqrt{\lambda }}} &{} 0 \\ 0 &{} 0 &{} \lambda \end{array} \right) ,\nonumber \\ {\varvec{B}}= & {} \hat{{\varvec{B}}} = {\varvec{C}} = \hat{{\varvec{C}}} = \left( \begin{array}{ccc} {\frac{1}{\lambda }}&{} 0 &{} 0 \\ 0 &{} {\frac{1}{\lambda }} &{} 0 \\ 0 &{} 0 &{} \lambda ^2 \end{array} \right) , \end{aligned}$$

with $$J=1$$.

A pure shear wave which will perturb the macro-deformation ${\varvec{U}}$ can be written as

13 $$\begin{aligned} \varvec{u}_{{c}}= \left( \begin{array}{ccc} u_1(x_2,x_3) \\ u_2(x_1,x_3) \\ u_3(x_1,x_2) \end{array} \right) . \end{aligned}$$

This form ensures that $\nabla \varvec{u}_{{c}}$ has only off-diagonal components, i.e. $\nabla \varvec{u}_{{c}}: {\varvec{I}} = 0$, thus satisfying the incompressibility assumption of the material. Since the deformation considered employs no shearing (i.e. no off-diagonal components), then also $\varvec{B}: \nabla \varvec{u}_{{c}}= 0$ and therefore this wave form allows for simplifications when being contracted, as needed in Eq. 9a, with the stiffness modulus by removing the terms containing $\delta _{ml}$ and ${\hat{B}}_{ml}$ from Eq. 11:

$$\begin{aligned} \varvec{\mathcal {G}} ^\prime : \nabla \varvec{u}_{{c}}=\frac{1}{J} \Big [ \mu _e \nabla \varvec{u}_{{c}}{\varvec{B}} + \nabla \varvec{u}_{{c}}^T \Big (\frac{\mu _e I_{{\hat{\varvec{B}}}}}{3} -PJ \Big ) \Big ]. \end{aligned}$$

Additionally, under the divergence operator, $\nabla \varvec{u}_{{c}}^T$ vanishes (together with $I_{{\hat{\varvec{B}}}}, \,P$ and *J*, which are constant in space), and thus, the elastic contribution can be further simplified to

14 $$\begin{aligned} \nabla \cdot ( \varvec{\mathcal {G}} ^\prime : \nabla \varvec{u}_{{c}}) = \nabla \cdot (\mu _e \nabla \varvec{u}_{{c}}{\varvec{B}}) \end{aligned}$$

Let us now analyse particular examples of shear waves—namely planar waves—travelling through the compressed cuboid. In the first case, consider that the front face of the cuboid is vibrated along the ${\varvec{e}}_1$ direction (see Fig. 1), an idealised wave of the form

15 $$\begin{aligned} \varvec{u}_{{c}}= \left( \begin{array}{ccc} u_1(x_2)\\ 0 \\ 0 \end{array} \right) \end{aligned}$$

is created, with the only nonzero entry in the wave displacement gradient being $(\nabla \varvec{u}_{{c}})_{12}$. Therefore, the deformation contribution in Eq. 14 simply becomes a scaling by $1/\lambda$:

$$\begin{aligned} \mu _e \nabla \varvec{u}_{{c}}{\varvec{B}} = \frac{\mu _e}{\lambda } \nabla \varvec{u}_{{c}}. \end{aligned}$$

As such, the loading bias is influencing the apparent stiffness of the cuboid, which appears to be $\mu _e/\lambda$, whereas the intrinsic stiffness is $\mu _e$. Thus, under this setup of the wave, the material stiffness appears to be higher, since $\lambda <1$.

In the second case, consider a planar wave created by vibrating the top face of the cuboid, this time along the ${\varvec{e}}_2$ direction (see Fig. 1). Then, the idealised wave displacements

16 $$\begin{aligned} \varvec{u}_{{c}}= \left( \begin{array}{ccc} 0 \\ u_2(x_3) \\ 0 \end{array} \right) \end{aligned}$$

are probing the cuboid in the direction in which it was originally compressed, since the only nonzero component of the wave gradient is $(\nabla \varvec{u}_{{c}})_{23}$. This results in a stiffness scaling by $\lambda ^2$, i.e.

$$\begin{aligned} \mu _e \nabla \varvec{u}_{{c}}{\varvec{B}} = \lambda ^2 \mu _e \nabla \varvec{u}_{{c}}. \end{aligned}$$

With $\lambda <1$, the biased stiffness $\lambda ^2 \mu _e$ appears to be softer than the material’s true stiffness $\mu _e$.

From analysing these two simple instances of planar waves (Eqs. 15 and 16), we gain an understanding of how the waves probing a material under compression experience different loading bias—stiffer in the expanded direction ${\varvec{e}}_{1}$ and softer in the compressed direction ${\varvec{e}}_3$. Had the waves been more complex, like in Eq. 13, then the effect of the loading would yield

$$\begin{aligned} \mu _e \nabla \varvec{u}_{{c}}{\varvec{B}} = \mu _e \left( \begin{array}{ccc} 0&{} \dfrac{1}{\lambda } \dfrac{\partial u_1}{\partial x_2} &{} \lambda ^2 \dfrac{\partial u_1}{\partial x_3} \\ \dfrac{1}{\lambda } \dfrac{\partial u_2}{\partial x_1} &{} 0 &{} \lambda ^2 \dfrac{\partial u_2}{\partial x_3} \\ \dfrac{1}{\lambda } \dfrac{\partial u_3}{\partial x_1} &{} \dfrac{1}{\lambda } \dfrac{\partial u_3}{\partial x_2} &{} 0 \end{array} \right) . \end{aligned}$$

Thus, even under simplified conditions like an idealised shear wave travelling through a Neo-Hookean material, we get a feeling for how biased the wave propagation becomes under pure compression. It can be presumed that, under less than ideal conditions (more complex materials and deformations), the harmonic wave motion becomes increasingly intricate.

###  Nonlinear viscoelastic characterisation of PVA

Experiments done in controllable media, where shapes and deformations are simple, constitute the first step in evaluating the theory explained in the previous section. A first key component is using a material with a known rheological behaviour, as this governs the effective loading bias (e.g. Eq. 6). The following sections describe the fabrication process of PVA material and its testing in a rheological setup. A viscoelastic material law is also formulated. This law will then be later (Sect. 2.3.2) integrated with deformation and MRE data.

#### PVA phantom preparation

A material suitable for our purposes should be able to withstand large deformations without rupturing and to have low wave attenuation. For this, polyvinyl alcohol cryogel (PVA-C) was selected, which is suitable for mimicking soft tissue properties and has a high MR signal (Surry et al. 2004; Sinkus et al. 2005a). Following a protocol similar to the one described in Xia et al. (2011), phantoms were created by mixing polyvinyl alcohol powder (P1763 Sigma-Aldrich Company Ltd., UK) with deionised water in a concentration of 7%. A magnetic stirrer was used for 2 h, in order to fully dissolve the powder into the water, while heating the concoction to 90$^{\circ }$C. The mixture was covered throughout the process, to avoid evaporation, left to cool down at room temperature and poured into cuboid moulds of dimensions $64 \times 48 \times 42 \, \hbox {mm}$. The moulds were then placed in a freezer at $-\,20^{\circ }$C and underwent three cycles of freezing (14 h) and thawing (10 h) (F-T). These specifications ensured that the material was suitable for testing under large deformations. Laboratory experimentation with higher PVA concentrations (e.g. 10%) led to phantoms that were not easily deformable, whereas reducing the number of F-T cycles led to unstable phantoms that would leak water under loading.

**Fig. 2:**
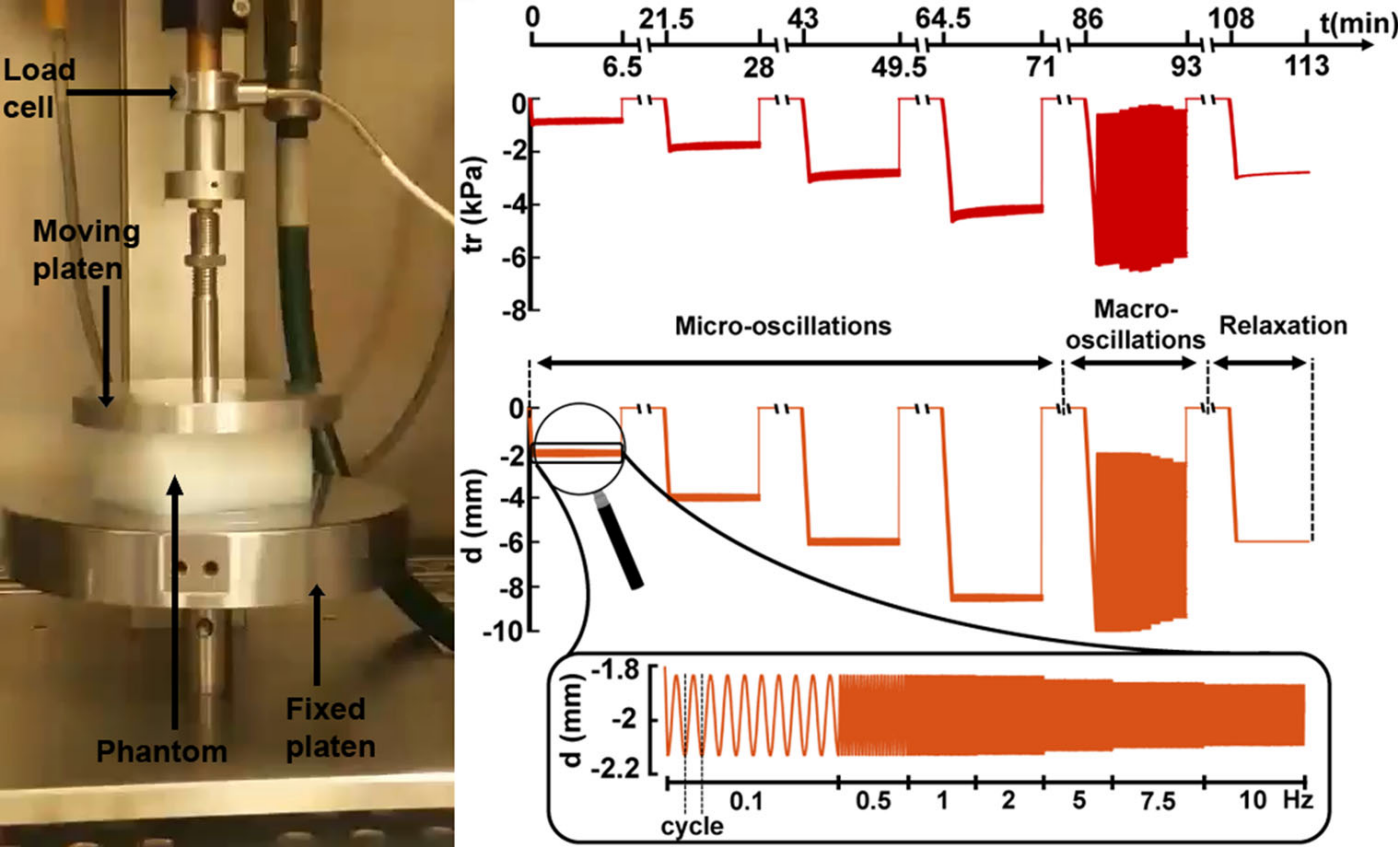
Illustration of experimental setup, protocol and data in rheological tests. (Left) Phantom in the rheological instrument. The moving platen is compressing the phantom and oscillates vertically, while the loading cell records the force. (Right, top) The traction measurements in one phantom for the six tests: four micro-oscillations and one macro-oscillation (sweeping over frequencies) and a relaxation test. (Right, bottom) Platen displacements, corresponding to phantom compression levels. Zoomed panel: exemplification of a micro-oscillatory test displacements, showing the frequency sweep; illustration of a cycle in the lowest frequency regime—one period, starting from the lowest point in the compression

During the freezing process, volumetric expansion occurred. Therefore, the phantoms’ top was cut, in order to achieve cuboid shapes. This led to differences in the phantoms’ height, which ranged between 32 and 42 mm. For a meaningful variance testing, 14 homogeneous phantoms were created, out of which 7 were used for rheological experiments and 7 for MRE experiments. However, one phantom was excluded in the rheological tests due to rupture. The time between phantom fabrication and testing in either rheology or MRE was within two weeks, to avoid potential material degradation.

#### Rheological testing of PVA phantoms

The phantoms allow for suitable examination methods that can test the theory developed in Sect. 2.1. It was seen that, in order to account for the nonlinear complex stiffness moduli $\varvec{\mathcal {G}} ^\prime$ and $\varvec{\mathcal {G}} ^{\prime \prime }$ (Eq. 10), understanding of the nonlinear behaviour and frequency dependence of the material must be inferred. This can be done through rheological testing, which is used to characterise material behaviour by quantifying its response to different types of applied stress. As such, each of the six phantoms created for rheology experiments was tested in a Bose Electroforce 5500 test instrument. The setup, seen in Fig. 2, consisted of a supporting fixed platen and an adjustable upper platen, which was movable in the vertical direction. A load cell of capacity 225N measured the force required to ensure a user defined deformation.

For our aims, three types of tests were employed, with the purpose of assessing PVA’s nonlinear behaviour. The first test was designed to investigate the material’s response to dynamic micro-oscillations around a state of large static load. This design replicates closely the in vivo MRE scenario, where low-amplitude harmonic waves are propagating in an organ which may be subjected to large deformation. For reproducing the nonlinear behaviour, four different static loading states were investigated. Thus, the moving platen was programmed to compress the phantom by 2, 4, 6 and 8 mm, respectively ($\sim$5–20% uniaxial compression), and then to oscillate with an amplitude of 0.15 mm ($\sim$0.4%). This test will be referred to as the micro-oscillatory test.

The second test was designed to investigate the nonlinear large deformation response in time. As such, large oscillations were imposed around a state of large deformation: the platen compressed the phantom by 5.8 mm ($\sim$15% uniaxial compression) and then oscillations of 4 mm ($\sim$10%) amplitude were imposed. This test will be referred to as the macro-oscillatory test.

In both tests, oscillatory tests were carried under a frequency sweep, from 0.1 Hz to 10 Hz (the instrument’s limit): 0.1, 0.5, 1, 2, 5, 7.5 and 10 Hz. A clear depiction of the testing protocol can be seen in Fig. 2. This was done to investigate the frequency response of PVA, with the aim of extrapolating it to the higher frequencies used in MRE. In all tests, but clearly noticeable in the macro-oscillatory one, the displacements’ amplitude is decreasing with increasing frequency. This is probably due to the instrument’s limitation which, in the faster frequency regime, cannot reach the instructed displacements.

At the higher frequencies ($\ge \, 5 \, \hbox {Hz}$), it was observed that the moving platen gains acceleration and introduces a bias on the data. As such, calibration data were acquired, where the same protocol was followed, but with no sample in between the platens. Subtracting the calibration data from the phantom data eliminated the platen’s momentum bias.

A final stress relaxation test was conducted by subjecting the material to a constant large deformation, in order to capture the specific viscoelastic effect. Hence, the phantoms were held under a compression of 11 mm ($\sim$28.5%) for 5 min.

Overall, each phantom underwent six tests: four micro-oscillations at different loads (with seven different oscillatory states acquired), one macro-oscillation (with seven different oscillatory states acquired) and one relaxation. In between tests, the phantoms were left to rest in water for 15–20 min, since PVA is best stored in water (Surry et al. 2004), for hydration reasons. This resting time allowed for an efficient testing protocol of the phantoms, and its duration was sufficient to observe reproducibility.

#### Constitutive modelling of PVA

PVA and other hydrogels have been modelled, previously, as hyperelastic materials, employing laws that are generally used to describe rubber-like materials. Often, a Mooney–Rivlin type of model, employing two parameters, was found to be sufficient to capture the elastic behaviour of PVA (Anseth et al. 1996; Pazos et al. 2009). Extension to viscoelasticity was sometimes accomplished by considering Maxwellian elements (Anseth et al. 1996; King et al. 2011). As such, here the aim is to model the PVA material as viscoelastic, yet using springpot elements. Following previous investigations, we start the modelling process by considering the elastic part to be defined by a Mooney–Rivlin type of material law:

17 $$\begin{aligned} W_e(\varvec{F}) = C_1 ( I_{{\hat{\varvec{C}}}} - 3 ) + C_2 ( II_{{\hat{\varvec{C}}}} - 3 )^2, \end{aligned}$$

where $C_1$ and $C_2$ (Pa) are material parameters. Considering the definition of the elastic part of the PK2 stress given, generally, as ${\varvec{S}}_{e} = 2\nabla _{\varvec{C}}W_e$, it follows that ${\varvec{S}}_e$ has two elastic parts scaled by the material parameters $C_1$ and $C_2$:

$$\begin{aligned} {\varvec{S}}_e (\varvec{C}) = C_1 {\varvec{S}}_{e1} + C_2 {\varvec{S}}_{e2}. \end{aligned}$$

The elastic terms are derived to be

$$\begin{aligned} {\varvec{S}}_{e1}= & {} \dfrac{2}{J^{2/3}} \left( {\varvec{I}} - \dfrac{I_{\varvec{C}}}{3}\varvec{C}^{-1} \right) , \\ {\varvec{S}}_{e2}= & {} \dfrac{8}{J^{4/3}} \big ( II_{{\hat{\varvec{C}}}} - 3 \big ) \bigg ( \varvec{C}- \dfrac{II_{\varvec{C}}}{3}\varvec{C}^{-1} \bigg ). \end{aligned}$$

As previously mentioned in Sect. 2.1.2, we consider the viscous part of PK2 to depend on a fractional-order derivative of a viscoelastic stress ${\varvec{S}}_v$, which is defined here as the sum $\delta _1 {\varvec{S}}_{e1} + \delta _2 {\varvec{S}}_{e2}$. Hence, the PK2 tensor becomes

19 $$\begin{aligned} \varvec{ S} = C_1 \varvec{ S}_{e1} + C_2 \varvec{S}_{e2} + D_t^\alpha (\delta _1 \varvec{S}_{e1} + \delta _2 \varvec{S}_{e2} ) + {\varvec{S}}_{p}. \end{aligned}$$

Here, $\alpha$ is the fractional derivative coefficient, with $\alpha =0$ preserving the viscoelastic stress ($D_t^\alpha {\varvec{S}}_e = {\varvec{S}}_e$), which turns into a purely elastic contribution, and $\alpha =1$ indicating the first-order derivative, which becomes a purely viscous contribution. The hydrostatic stress ${\varvec{S}}_p = J P {\varvec{C}}^{-1}$ completes the definition of our PK2 tensor.

#### Rheology data analysis and model fitting

The viscoelastic model derived in the previous section (Eq. 19) needs to be tailored to the PVA material used in the rheological experiments by finding appropriate parameters $C_1, \, C_2, \, \delta _1, \, \delta _2$ (Pa) and $\alpha$.

As previously mentioned, the acquired rheological data consist of force measurements required to ensure a predefined displacement, each corresponding to a point in time. This was transformed into traction data in the $\varvec{e}_3$ direction by dividing the force readings by the cuboid’s top area adjusted to the deformation, i.e.

$$\begin{aligned} tr^d(t) = \frac{F(t)}{A(t)}, \quad A(t) = \frac{A_0 h_0}{h(t)}. \end{aligned}$$

Here, $tr^d(t)$ is the traction obtained from the data, *F*(*t*) is the force reading, and *A*(*t*) is the area at time *t*, which depends on the initial top area $A_0$ and the ratio of initial cuboid height $h_0$ to the compressed height *h*(*t*) (with $h(t) = h_0 - d(t)$).

Likewise, the traction in the ${\varvec{e}}_3$ direction arising from the model ($tr^m$) was computed using

$$\begin{aligned} tr^m(t) = ({\varvec{\sigma }}(t) \cdot {\varvec{n}})_3, \quad {\varvec{\sigma }} = {\varvec{F}} {\varvec{S}} {\varvec{F}}^T, \end{aligned}$$

where ${\varvec{\sigma }}$ is the Cauchy stress and ${\varvec{n}}$ is the normal to the surface (in our case, we are interested in the normal to the top surface, i.e. ${\varvec{n}} = [0, \, 0, \, 1]^T$). Since the deformation at any point in time is given by

$$\begin{aligned} {\varvec{U}}(t) = \left( \begin{array}{ccc} \left( \sqrt{\frac{h(t)}{h_0}}-1 \right) X_1 \\ \left( \sqrt{\frac{h(t)}{h_0}}-1 \right) X_2 \\ \left( \frac{h_0}{h(t)}-1 \right) X_3 \end{array} \right) , \end{aligned}$$

the elastic and viscoelastic tensors ${\varvec{S}}_{e1,2}$ and $D_t^\alpha {\varvec{S}}_{e1,2}$ can be determined, together with their counterparts in the Cauchy stress. They take the form of diagonal second-order tensors. The hydrostatic pressure *P* can also be determined at each time point by noticing that the traction in the ${\varvec{e}}_{1,2}$ directions is 0. Hence, *P* counteracts the elastic and viscoelastic contributions in both the ${\varvec{e}}_{1,2}$ directions and can be used as such in computing the traction in the ${\varvec{e}}_3$ direction. Thus, the only unknowns in the traction model are parameters $C_1, \, C_2, \, \delta _1, \, \delta _2, \, \alpha$. In broad terms, since $\alpha$ is the only nonlinear parameter of the model, the parameterisation process is done by iterating over fixed values of $\alpha$ and then solving a minimisation problem which yields a best fit of the remaining linear parameters. It is desired that all tests (micro-, macro-oscillations and relaxation) carry the same importance in the fitting process, and that the error is not dominated by certain tests (e.g. those done at higher compression levels, which employ larger tractions). As such, for each of the six tests (four micro-oscillation, one macro-oscillation and one relaxation), the traction data and model were standardised according to the maximum traction value in the test, i.e.

$$\begin{aligned} tr^d(t) = \frac{tr^d(t)}{\underset{t}{\max }|tr^d(t)|} , \qquad tr^m(t) = \frac{tr^m(t)}{\underset{t}{\max }|tr^d(t)|} \end{aligned}$$

where $tr^d(t)$ and $tr^m(t)$ are kept for simplicity, to avoid addition of further notation.

Three characteristics were considered to be important when fitting the model to the data: the compression, relaxation and oscillatory response. Thus, the error to be minimised was designed to comprise two parts, the first one dealing with the compression and relaxation behaviour, and the second one with the oscillatory behaviour. For the first part, in order to ensure that the error is not dominated by compression only, but also by relaxation, it is important to consider the data broken down into cycles, i.e. one full oscillation starting from the lowest load state, equivalent to one period (see Fig. 2). Each of these cycles is confined within a generic time interval $[t_1,t_2]$. A mean traction value (${\bar{tr}}^d$, ${\bar{tr}}^m$ for data and model, respectively) is defined as the integral of the traction over the cycle, e.g.

$$\begin{aligned} {\bar{tr}}_k^d = \int _{t1}^{t2} tr^d(t) \text { d}t, \quad {\bar{tr}}_k^m = \int _{t1}^{t2} tr^m(t) \text { d}t, \end{aligned}$$

over generic cycle *k*. This mean value captures the compression level and, by considering locally every cycle in a test, captures the relaxation behaviour. Hence, the first part of the error deals with the sum, over all cycles, of the mean traction value difference between the data and the model, normalised by the data:

$$\begin{aligned} \text {err}_1 = \frac{\sum _k ({\bar{tr}}_k^d - {\bar{tr}}_k^m)^2}{\sum _k ({\bar{tr}}_k^d)^2}. \end{aligned}$$

The second part of the error is designed to measure the difference in the oscillatory behaviour between data and model. Thus, the traction amplitude corresponding to a generic time point $t_j$ in a cycle *k* is found as the difference between the traction value and the traction mean over the cycle, e.g.

$$\begin{aligned} tr^d(t_j^k) - {\bar{tr}}_k^d, \qquad tr^m(t_j^k) - {\bar{tr}}_k^m. \end{aligned}$$

The difference in amplitudes between the model and the data is investigated across all time points in a cycle, over all cycles, and is subsequently normalised by the data, as

$$\begin{aligned} \text {err}_2 = \frac{\sum _k \sum _j \big [ (tr^d(t_j^k) - {\bar{tr}}_k^d) - (tr^m(t_j^k) - {\bar{tr}}_k^m) \big ]^2}{\sum _k \sum _j \big ( tr^d(t_j^k)-{\bar{tr}}_k^d \big ) ^2}. \end{aligned}$$

We note that the standardisation of the data was done over each of the six tests separately, to ensure that they carry equal importance within the error, while the normalisation was done over the tests altogether, to facilitate the interpretation of the error, which is finally outlined as

20 $$\begin{aligned} \text {err} = \sqrt{\frac{\text {err}_1 + \text {err}_2}{2} }. \end{aligned}$$

This form yields 0% for a perfect fit and 100% when $C_1, \, C_2, \, \delta _1, \, \delta _2 = 0$ Pa, irrespective of $\alpha$.

Having defined the error to be minimised, a more detailed parameter fitting process can be described. The nonlinear parameter $\alpha$ is iterated between 0.05 and 1 (close to the elastic and viscous limits, respectively), with an incremental step of 0.05. Since the PVA material used has a low viscoelastic response (and $\alpha =0.05$ was observed to be the most suitable), the incremental step was refined to be 0.01 between 0.01 and 0.1. For each fixed $\alpha$, the linear parameters $C_1, \, C_2, \, \delta _1, \, \delta _2$ were searched by solving the least squares problem corresponding to error Eq. 20, using the inbuilt function *lsqnonlin* of MATLAB and Statistics Toolbox Release 2015a, The MathWorks, Inc., Natick, Massachusetts, USA. The nonlinear solver was used in order to allow for a positivity constraint on the parameters. For each phantom, a full set of parameters was determined.

To ensure parameter uniqueness, once the best $\alpha$ value was determined, a new parameter search was performed. Thus, each individual linear parameter ($C_1, \, C_2, \, \delta _1 \text { and } \delta _2$, respectively) was fit using the process described above, but while imposing the remaining three linear parameters to be 0 Pa. This was done in order to gauge the value of each model component and to identify potential parameter coupling.

### Harmonic wave motion in deformed PVA

After characterising the PVA material through a viscoelastic model, the next essential element in estimating the intrinsic material parameters and bypassing the loading bias is merging the information on deformation and rheology with MRE data. Thus, in what follows, the MRE experimental features used to obtain simple deformations are described. The core component of integrating material behaviour and deformation knowledge into the data analysis process is outlined.

**Fig. 3:**
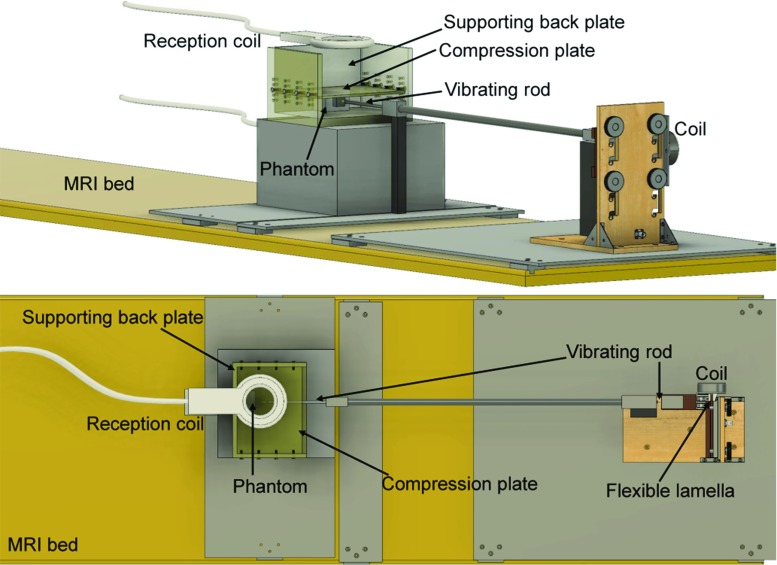
Illustration of the MRE test setup. (Top) 3D view of the setup. A coil vibrating to the frequency established by the MRE sequence is connected to a flexible lamella, which causes an attached rod to vibrate longitudinally. A piston fit at the end of the rod is indenting the phantom, thus generating compressional waves. The phantom is resting on a smooth support and is in contact with the back support plate, which prevents the phantom from slipping and thus helps converting the compressional waves into shear waves. An upper plate compressing the phantom is kept in place by bolts fixed to the side plates. Reception coils are placed around the phantom, for signal enhancement. (Bottom) 2D top view of the setup

**Fig. 4:**
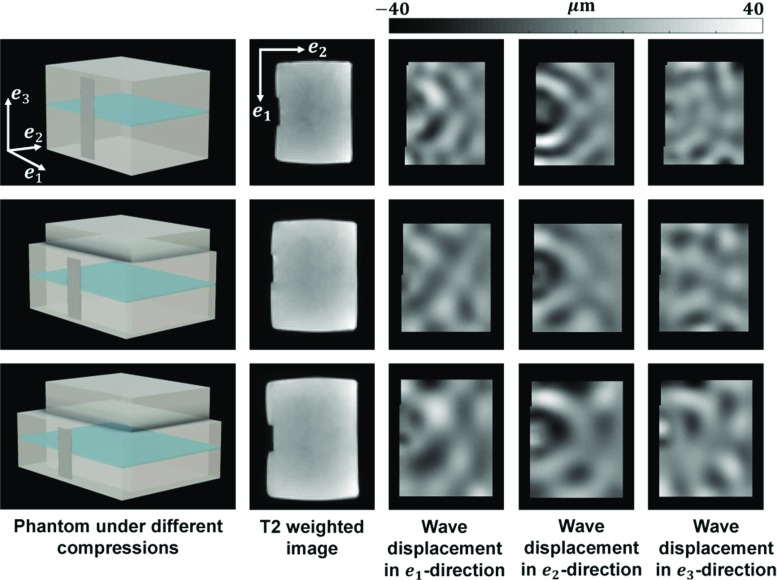
Images corresponding to the uncompressed (top row) and compressed (middle and bottom rows) phantoms (here illustrated in phantom 12). (First column) Phantom depicted at the different deformation states. The uncompressed state is kept for reference. The piston (grey bar) is indenting the phantom perpendicularly during the MRE scan. Slices are acquired in the coronal plane (depicted in blue). (Second column) Cross-sectional (coronal) view from the T2 weighted images. The piston indentation can be seen, as well as the expansion of the phantom in the ${\varvec{e}}_1-{\varvec{e}}_2$ directions under compression. Wave displacements from the MRE imaging protocol can be seen in the ${\varvec{e}}_1$ direction (third column), in the ${\varvec{e}}_2$ direction (fourth column) and in the ${\varvec{e}}_3$ direction (fifth column). It can be observed in the wave displacements, between the three rows, that the wavelength increases under compression. The wave images in the third, fourth and fifth columns have been cropped around the phantom area, to exclude the surrounding noise

#### MRE experimental setup

When designing the MRE experiment, it was important to obtain shear waves that would propagate through the whole body of the phantom, while maintaining a fixed position of the phantom. The setup used is illustrated in Fig. 3. A transducer indenting the phantom transmits small amplitude compressional waves. Since the PVA material is aqueous, it could easily slide on a flat surface. For this reason, the phantom’s back side was in contact with the support, ensuring no penetration, hence converting the compressional waves into shear waves. A large uniaxial deformation could be obtained by simply compressing the phantom with a smooth top plate, as seen in Fig. 3. Due to the sliding feature of the PVA material, the phantoms did not bulge under the uniaxial compression just described.

Using this setup inside the MR scanner, coronal wave data were recorded using MRE in undeformed and deformed configurations. Each phantom was scanned in the reference state (uncompressed), first deformation state consisting of uniaxial compression of $\sim$5 mm ($\sim$14%) and second deformation state consisting of uniaxial compression of $\sim$12 mm ($\sim$35%).

MR acquisition consisted of two primary scans. First, a geometric scan was acquired providing 55 cross-sectional slices with a field of view (FOV) of $96 \times 96 \, \hbox {mm}$ (resolution of $96 \times 96$ pixels and slice thickness of 1 mm). Scan parameters were adjusted to enhance signal of the PVA material (TE/TR= 120.00 /5000.00 ms).

The second scan was a MRE eXpresso Gradient Echo sequence (Garteiser et al. 2013) providing 10 cross-sectional slices with a FOV of $96 \times 96 \, \hbox {mm}$ (resolution $96 \times 96$ pixels and slice thickness of 2.0 mm). Driver frequency and motion encoding gradients were set, by turn, at 120, 130 and 140 Hz, and TE/TR at 6.91 /151.82 ms. The sequence encoded each of the three displacement component direction—$\varvec{e}_1$, $\varvec{e}_2$, $\varvec{e}_3$ and a reference non-motion encoded image removing background phase shifts due to the MRI gradients. Examples of the geometric and wave images can be seen in Fig. 4, for the undeformed and deformed configurations.

#### MRE data analysis

The geometric data acquired were used to provide detailed quantitative information of the geometrical configuration of the material in reference and deformed states. Assuming that the cuboid phantoms were compressed uniformly, then the deformation gradient can be written as in Eq. 12, where $\lambda$ is the compression defined as the ratio of the compressed and uncompressed heights. Hence, due to the design of the MRE experiment, this form of the deformation gradient can be employed with the stiffness moduli given by Eq. 10.

From the recorded small amplitude displacements, two reconstruction methods were used. Firstly, a divergence free finite element reconstruction solving the set of Eq. 9 (Fovargue et al. 2018a) was used to retrieve the stiffness of the PVA phantom, merging the datasets acquired at 120, 130 and 140 Hz, for a better signal to noise ratio. This method takes as input the material density $\rho$, frequency $\omega$ and wave displacements $\varvec{u}_{{c}}$ and reconstructs the complex stiffness modulus $G^*$ by assuming

21 $$\begin{aligned} \varvec{\mathcal {G}} ^* = G^*\varvec{\mathcal {I}} . \end{aligned}$$

**Fig. 5:**
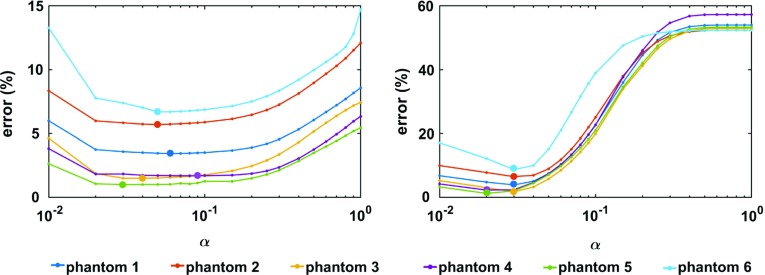
The minimum error (per Eq. 20) obtained by fitting the model to the data for each phantom, sweeping over fixed values of $\alpha$ between 0.01 and 1. (Left) The error for the 5-parameter model. (Right) The error for the 3-parameter model. The minimal error for each phantom, obtained by employing the parameters in Table 1 (5-parameter model) and Table 3 (3-parameter model), is enhanced

This is usually separated into the (real) storage modulus $G^{\prime }$ and (imaginary) loss modulus $G^{\prime \prime }$ (where $G^* = G^{\prime } + i G^{\prime \prime }$).

As explained in Sect. 2.1.2, the form of $\varvec{\mathcal {G}} ^*$ presented in Eq. 21 holds true in the absence of deformation and under an isotropic standard material law, and the wave gradient contribution becomes symmetric, as $\varvec{{\mathcal {I}}} : \nabla \varvec{u}_{{c}}= \nabla \varvec{u}_{{c}}+ \nabla \varvec{u}_{{c}}^T$. Hence, this method is suitable for reconstructing the stiffness modulus when no deformation is employed, but will lead to a biased result under deformation. Throughout the rest of the paper, this method will be referred to as uncorrected reconstruction (UR).

Secondly, the same reconstruction was adapted here, by integrating the formal definition of the real and imaginary stiffness moduli $\varvec{\mathcal {G}} ^{\prime }$ and $\varvec{\mathcal {G}} ^{\prime \prime }$ (Eq. 10) in order to account for the bias in apparent stiffness introduced by the deformations. Conceptually, instead of relying solely on the wave displacements and its gradient $\nabla \varvec{u}_{{c}}$, the reconstruction integrates the scaling due to large deformations into the wave displacements gradient. Hence, instead of solving for ${G}^{*}$, a modified form of the equations was developed, given below:

22a $$\begin{aligned}&-\rho \omega ^2 \varvec{u}_{{c}}- \nabla \cdot [{M} (\varvec{\mathcal {G}} ^\prime : \nabla \varvec{u}_{{c}}) ] \nonumber \\&-i \nabla \cdot [ ({N}(\varvec{\mathcal {G}} ^{\prime \prime } : \nabla \varvec{u}_{{c}}) ] - \nabla \cdot p_{{c}}\varvec{I} = \varvec{0}, \end{aligned}$$

22b $$\begin{aligned}&\quad \nabla \cdot \varvec{u}_{{c}}= 0, \end{aligned}$$

where a solution is sought for $$ M + i N$$. This second approach will be referred to as the corrected reconstruction (CR).

As previously mentioned, within the UR the wave gradient is contracted with the fourth-order identity tensor scaled by the complex stiffness modulus, as $(G^* \varvec{\mathcal {I}} ):\nabla \varvec{u}_{{c}}$. By contrast, the CR allows the wave to be contracted with two distinct fourth-order tensors, defined by the user. One result is to be integrated with the real part and one with the imaginary part of the complex stiffness modulus, as ${M} (\varvec{\mathcal {G}} ^\prime : \nabla \varvec{u}_{{c}}) + iN (\varvec{\mathcal {G}} ^{\prime \prime } : \nabla \varvec{u}_{{c}})$, where $M+iN$ is to be found. This is a novel way of analysing MRE data and is of utmost importance, since we saw, in Eq. 10, that the real and imaginary moduli $\varvec{\mathcal {G}} ^{\prime }$ and $\varvec{\mathcal {G}} ^{\prime \prime }$ are non-trivial and change differently with deformation. These changes depend on metrics derived from the known deformation gradient ${\varvec{F}}$ (${\varvec{B}}$, $I_{{\varvec{C}}}$, etc.), and, for our PVA material, are scaled by the parameters determining the viscoelastic Eq. 19. The specific form can be found in Appendix 2, which is applicable to the PVA material used here. Thus, assuming that the viscoelastic model describes the data well, then $\varvec{\mathcal {G}} ^{\prime }$ and $\varvec{\mathcal {G}} ^{\prime \prime }$ already incorporate the true real and imaginary stiffness moduli. Hence, it is predicted that *M* and *N* will be reconstructed as unity.

With this understanding of the newly developed CR, the PVA material model was parameterised, this time employing the MRE data. The reference and second compression states were used, and the left hand side of Eq. 22a was minimised over all pixels. That is, the linear model parameters were sought such that *M* and *N* were reconstructed as closely as possible to unity for each pixel, over all pixels. The nonlinear parameter $\alpha$ was fixed to be the one indicated by rheology tests. The set of parameters thus obtained from the reference and second compression states was tested against the first compression state, for prediction value. The error was measured as the pixel-wise difference between the reconstructed values *M* and unity in uncompressed and second compression case, normalised by the number of pixels, as

23 $$\begin{aligned} \text {err} = \sqrt{\frac{\sum _{k=1}^n \left( M_k - 1 \right) ^2}{n}}, \end{aligned}$$

where *n* is the total number of pixels considered and subscript *k* iterates over those pixels.

## Results and discussion

The first essential step in the experimental work was tailoring the viscoelastic law 19 to the PVA phantoms used in the rheology testing. The results of this process are presented in Sect. 3.1 and help us understand the rheological behaviour of the PVA material used, which directly influences the loading bias. The deformation bias is shown in Sect. 3.2, by investigating uniaxial compression in the MRE experimental setup. In Sect. 3.3 it is shown that, by incorporating the information on rheology and deformation into the CR, the intrinsic stiffness of the PVA material can be retrieved from the loaded states of the phantoms.

### Nonlinear viscoelastic model for PVA

The aim of the modelling process was to fit the viscoelastic model 19 to the PVA data acquired in the rheological setup. Hence, for each of the six phantoms used in the rheology experiment, the parameters were fit accounting for all oscillatory and relaxation tests simultaneously. It will be seen that the parameters cannot be uniquely identified, which stems from the fact that the PVA has a small viscoelastic response, as it will be explained shortly. As such, a model with a reduced number of parameters is sought, which ensures unique parameter estimation.

**Fig. 6:**
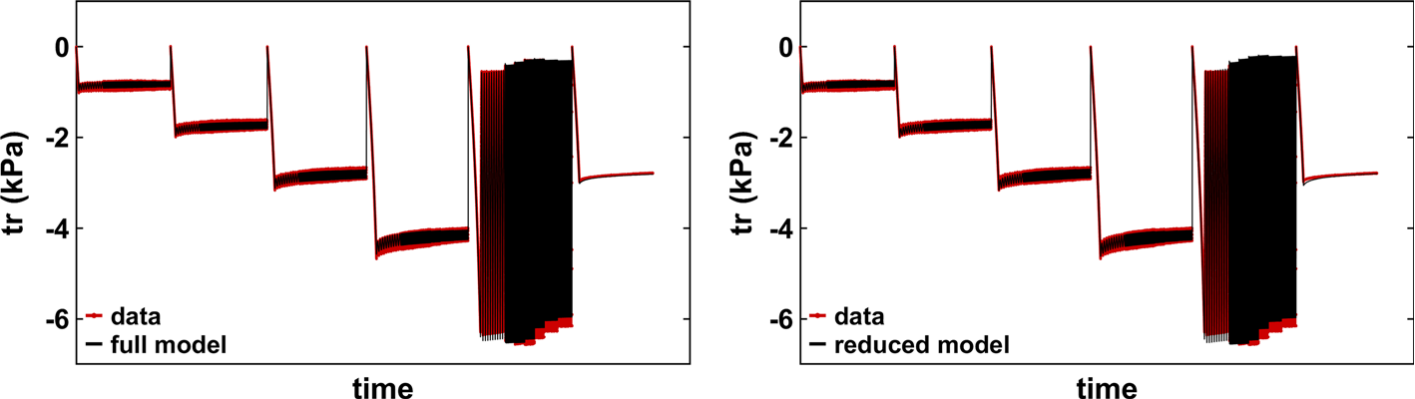
Illustration of the model fit considering the 5-parameter model or 3-parameter model in phantom 3. (Left) The model fit to the data using all parameters: $C_{1}$, $C_2$, $\delta _{1}$, $\delta _2$ and $\alpha$ (with an error of 1.50%). (Right) The model fit to the data using a reduced number of parameters: $C_2$, $\delta _{1}$, and $\alpha$ (with an error of 1.78%). The parameter values can be seen in Tables 1 and 3

Initially, the 5-parameter model described by Eq. 19 was fit to the data, considering $\alpha$ values between 0 and 1. Figure 5 (left) depicts the errors for each of the six phantoms used in rheology. All phantoms yield a minimal error around an $\alpha$ value of 0.03–0.09. The minimal error increases only slightly at higher $\alpha$ values. The reason for the shallow changes in minimal error at high $\alpha$ values is that the 2 viscoelastic parameters $\delta _1$ and $\delta _2$ become very small; hence, the data are fit mostly with the elastic parameters $C_1$ and $C_2$. Due to the PVA material having a small viscoelastic response, employing only an elastic model is not highly detrimental to the model fit, hence the small errors even at $\alpha =1$. The best parameter fit for each phantom is summarised in Table 1. The errors are small (less than $\sim$7%), which indicates that a good fit is ensured for each test. An example of the model fit can be seen in Fig. 6 (left). It can be observed that the compression level and relaxation behaviour are properly captured, while micro-oscillatory amplitudes are slightly underestimated. Notably, a large variability can be seen for all parameters with standard deviations around the mean spanning more than 100%. This can be explained by looking at the $\alpha$ parameter, which is generally small (0.03–0.09). These values indicate that the PVA has a very small viscoelastic response. Mathematically, if $\alpha =0$, then the linear parameters $\{ C_1, \delta _1 \}$ and $\{ C_2 , \delta _2 \}$ become pairwise redundant, since they scale the same component. Hence, with a very low $\alpha$, a coupling of the parameters arises. For instance, it can be seen, in Table 1, that the phantoms for which a high $\delta _1$ was yielded had a small $C_1$ and vice versa.

**Table 1:** Best fit parameters for each PVA phantom; the error, computed as per Eq. 20, can be seen in the first column (in brackets)

Phantom	$C_1$ (Pa)	$C_2$ (Pa)	$\delta _1$ (Pa)	$\delta _2$ (Pa)	$\alpha$
p1 (3.44%)	1545.66	0.18	195.76	149.40	0.06
p2 (5.69%)	1683.89	11.55	0.47	275.13	0.05
p3 (1.50%)	1606.03	4.50	1457.86	193.03	0.04
p4 (1.71%)	2491.84	57.71	628.60	79.40	0.09
p5 (1.01%)	647.09	174.31	2259.66	2.00	0.03
p6 (6.69%)	1746.13	0.04	0.00	272.97	0.05
Mean	1620.11	41.38	757.06	161.99	0.05
SD	537.34	62.71	839.25	98.87	0.019

**Fig. 7:**
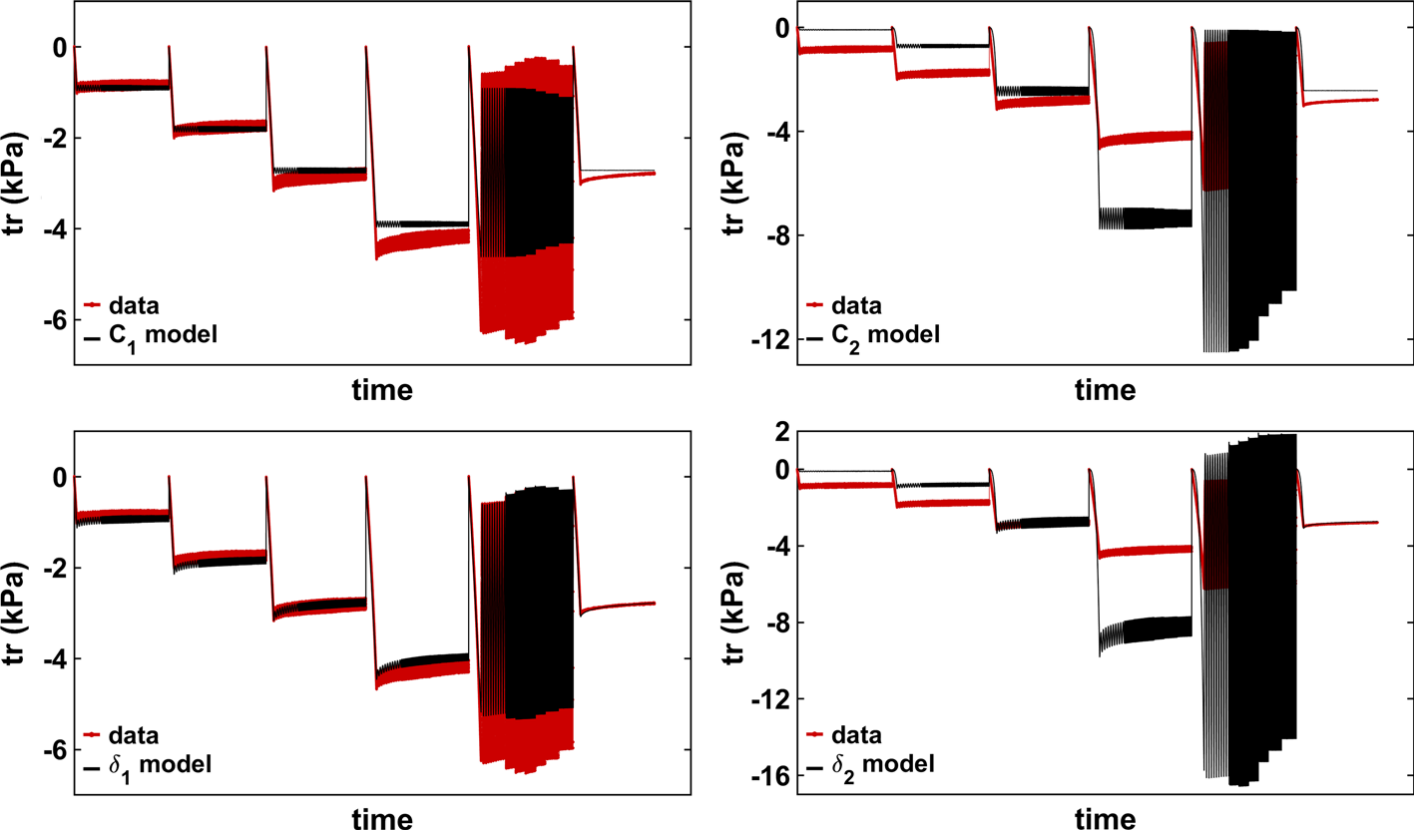
Contribution of each parameter to the model (black) compared to the data (red) (here, in phantom 4). Each of the four linear parameters ($C_{1}$ (top left), $C_2$ (top right), $\delta _{1}$ (bottom left), $\delta _2$ (bottom right)) was fit to the data, while setting the other 3 to be 0 ($\alpha$ was kept constant at 0.03). In each quadrant, the traction is depicted along the *y*-axis and the time along the *x*-axis (waiting times between tests not plotted for convenience). The parameters and errors can be seen in Table 2

Due to the small viscoelastic response of the PVA phantoms, parameters $\{ C_1, \delta _1 \}$ and $\{C_2, \delta _2\}$ tend to exhibit very similar features. As such, in order to identify each parameter contribution to the model, $\alpha$ was set to 0.03 (the best choice according to Fig. 5 (right)) and each of the linear parameters was fit individually. The effects of each individual parameter on the model are illustrated in Fig. 7. When considering only $C_1$, the model component considered is ${\varvec{S}}_{e1}$, which is equivalent to the Neo-Hookean elastic law. This parameter captures reasonably well the compression level of each test, indicating that the PVA material used has a dominant linearly elastic response. However, it lacks the ability to capture the changes in oscillatory amplitudes—in the four repetitions of the micro-oscillatory tests, the data indicates an increased amplitude at higher compression levels, whereas the model shows the same amplitude. The decrease in amplitude seen in the macro-oscillatory test is a direct result of the decrease in the actual displacement amplitude data, as seen in Fig. 7. This amplitude behaviour is entirely expected from the Neo-Hookean model, and the mismatch between the model and data indicates that the PVA displays nonlinear characteristics. Lastly, no relaxation behaviour is displayed, which is also expected due to the model component being purely elastic. Conversely, the fractional derivative of the ${\varvec{S}}_{e1}$ component, scaled by $\delta _1$, is the viscoelastic counterpart of the Neo-Hookean model. As such, investigating the value of $\delta _1$ leads to similar observations as for $C_1$, with the obvious difference that it can capture the relaxation behaviour. It is stressed, once again, that the similarity occurs due to $\alpha$ being very small.

**Table 2:** Best fit parameter for each PVA phantom; the error, computed as per Eq. 20, can be seen below each parameter; each parameter was fit considering the remaining three parameters to be 0

Phantom	$C_1$ (Pa)	$C_2$ (Pa)	$\delta _1$ (Pa)	$\delta _2$ (Pa)	$\alpha$
p1	1835.42	685.33	2175.25	813.40	0.03
err (%)	11.69	53.97	9.80	58.12	
p2	1933.73	884.40	2318.04	1031.81	0.03
err (%)	17.78	52.34	15.18	59.42	
p3	3015.56	1190.96	3568.47	1433.38	0.03
err (%)	9.29	52.93	7.31	56.10	
p4	3036.63	1072.38	3585.49	1258.29	0.03
err (%)	7.89	57.27	6.24	60.80	
p5	2864.40	1132.68	3374.09	1346.91	0.03
err (%)	8.70	53.30	8.70	56.92	
p6	1934.22	890.65	2363.49	922.69	0.03
err (%)	22.35	52.31	15.39	65.47	
Mean	2436.66	976.06	2897.47	1134.41	NA
SD	590.75	189.85	677.24	248.31	NA

When investigating $C_2$ , the nonlinearity of the ${\varvec{S}}_{e2}$ component becomes obvious—a linear increase in the compression level (2 mm between the micro-oscillatory tests) leads to a nonlinear response in the traction force. Hence, this parameter alone provides an unsuitable fit to the data; however, it adds value to the full model by being able to adjust for the increased amplitude in the four micro-oscillation tests. As before, the viscoelastic counterpart $\delta _2$ captures the relaxation behaviour. Table 2 presents the best fit parameter for each PVA phantom. By investigating Fig. 7, it is expected that the $C_1$ and $\delta _1$ parameters provide a better fit, and hence yield smaller errors than the $C_2$ and $\delta _2$ parameters.

Having looked at each individual component, it is reasonable to conclude that two out of the four linear parameters—one elastic and one viscoelastic non-counterpart—are sufficient for describing the PVA material behaviour. By keeping either $C_1$ or $\delta _1$, the compression level is ensured. By adding either $\delta _2$ or $C_2$, the amplitude fit can be improved for both the micro- and macro-oscillations. The relaxation is ensured by considering either viscoelastic component—$\delta _1$ or $\delta _2$, although from the individual parameter fit it looks like $\delta _1$ is more suitable for this aspect.

**Table 3:** Best fit parameters for each phantom, restricting $C_1, \delta _2 = 0$ Pa; the error, computed as per Eq. 20, can be seen in the first column, in brackets

Phantom	$C_2$ (Pa)	$\delta _1$ (Pa)	$\alpha$
p1 (3.95%)	118.94	1923.03	0.03
p2 (6.54%)	233.16	1900.25	0.03
p3 (1.78%)	169.10	3220.10	0.03
p4 (2.32%)	117.86	3143.31	0.02
p5 (1.28%)	176.42	2854.77	0.02
p6 (8.74%)	216.15	1973.74	0.03
	Linear	Linear	Nonlin
Mean	171.94	2502.53	0.03
SD	43.71	581.34	0.005

These observations indicate that the best parameters to fit are $C_2$ and $\delta _1$, while setting $C_1$ and $\delta _2$ to 0 Pa. Indeed, when investigating all pairwise tests (i.e. fitting either $\{C_1, \delta _1\}$, $\{C_1 , \delta _2\}$, $\{C_2 , \delta _1\}$ or $\{C_2 , \delta _2\}$, with $\alpha =0.03$), the smallest errors were obtained when considering $C_2$ and $\delta _1$. Hence, the initial model was reduced to 3 parameters—$C_2, \, \delta _1 \text { and } \alpha$-, with $\delta _1$ modelling the Neo-Hookean viscoelastic component, and $C_2$ spanning the nonlinear behaviour. The simplified PK2 tensor that characterises the PVA material becomes

24 $$\begin{aligned} \varvec{ S} = C_2 \varvec{S}_{e2} + \delta _1 D_t^\alpha \varvec{S}_{v1} + {\varvec{S}}_{p}. \end{aligned}$$

**Fig. 8:**
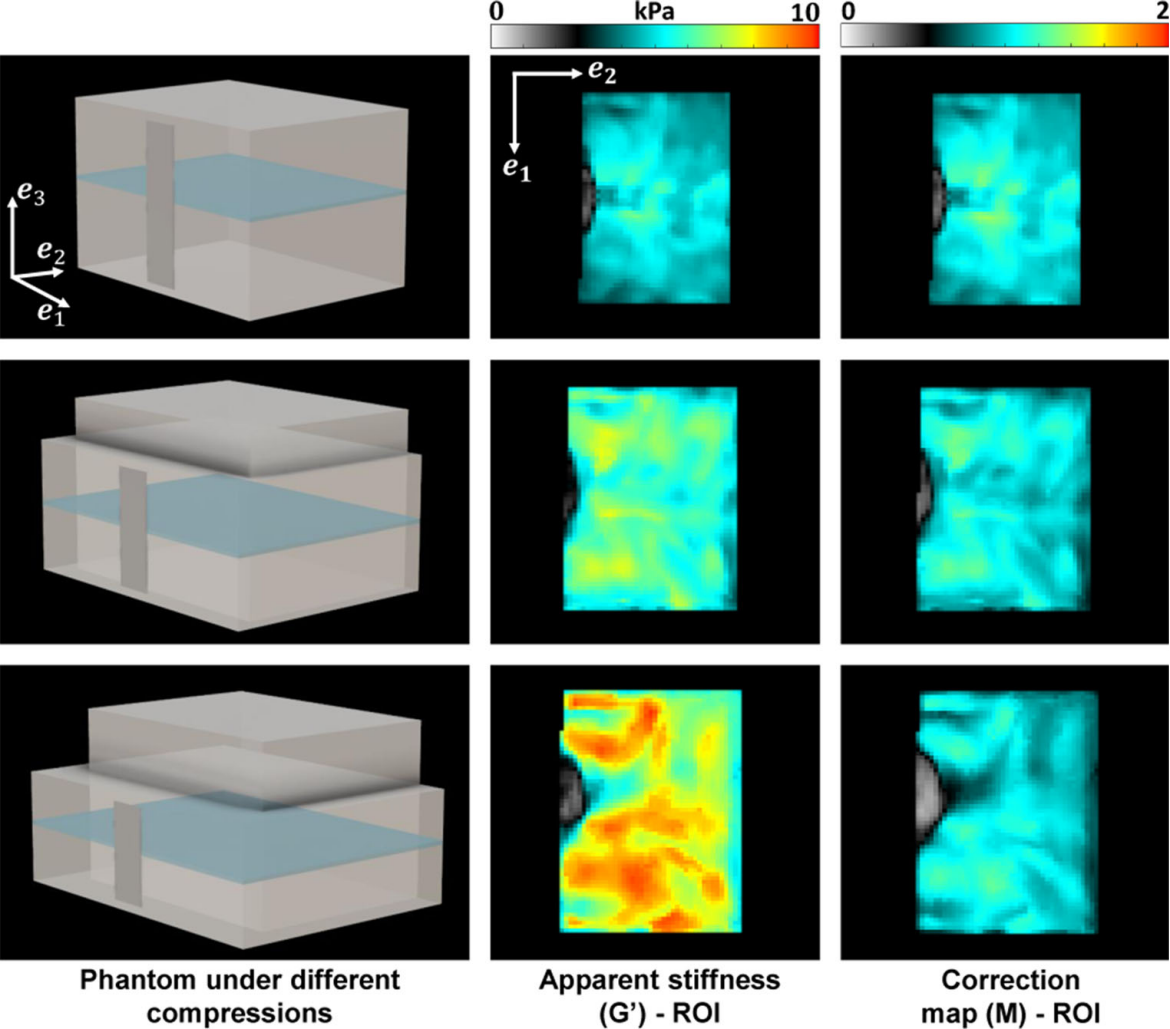
Stiffness maps $G^{\prime }$ and corrected maps *M* corresponding to the uncompressed (top row) and compressed (middle and bottom rows) phantoms (here illustrated in phantom 12). (First column) Phantom depicted at the different deformation states. The uncompressed state is kept for reference. The piston (grey bar) is indenting the phantom perpendicularly during the MRE scan. Slices are acquired in the coronal plane (depicted in blue). (Second column) Stiffness estimates using the UR. The region of interest (ROI) of the apparent stiffness map $G^{\prime }$ was obtained by eroding the phantoms’ margins. (Third column) Estimates of map *M* using the CR. The ROI of maps *M* was obtained by eroding the phantoms’ margins

The new set of parameters obtained for the 3-parameter model can be seen in Table 3. It is notable that the error increase is very small; thus, the new parametrisation does not significantly deteriorate the quality of the fit. An example of the parameterised 3-parameter model fit to the rheology data can be seen in Fig. 6 (right). The error plot of the 3-parameter model can be seen in Fig. 5 (right). Each phantom indicated a best fit for a very small $\alpha$ (0.02 or 0.03). At low $\alpha$ values, the errors are generally small, since the PVA is only slightly viscoelastic. However, at high $\alpha$ values, there is a sharp increase in the minimal error, which eventually plateaus. This happens when the contribution of the viscoelastic parameter becomes insignificant ($\delta _1$ becomes very small, to counteract the effect of $\alpha$) and the only remaining parameter is thus $C_2$. In this case, as seen in Table 2 as well, the errors rise up to $\sim$30–35%. In the low $\alpha$ regime, employing the 3-parameter model increases the errors only slightly compared to the 5-parameter model, which is preferred due to reduced complexity.

### Loading bias in harmonic wave motion under pure compression

In this study, the PVA phantom material is described by the moderately complex viscoelastic Eq. 24. Harmonic waves were designed to probe the PVA, as shown in Fig. 4 (columns 3–5).

**Fig. 9:**
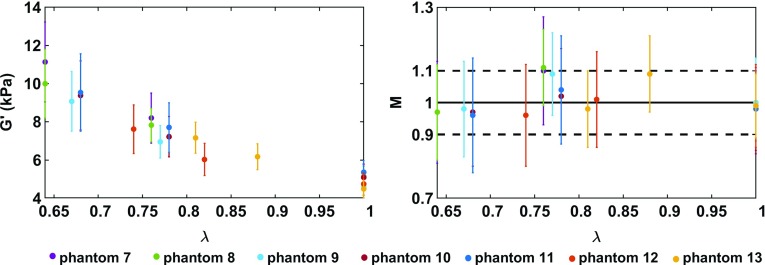
Average of stiffness maps $G^\prime$ and corrected maps *M* over all pixels in a phantom (excluding boundaries), in all deformation states. (Left) Average of stiffness map $G^\prime$ obtained using UR (Eq. 9) on the MRE data. In undeformed state, when $\lambda =1$, the average stiffness of the phantoms lies within 4.5-5.3 kPa. With increased compression, the phantoms appear to be stiffer, following a nonlinear trend. (Right) Average of corrected maps *M*, obtained by employing CR (Eq. 22) on the MRE data. The average value of the corrected maps *M* spans between 0.96 and 1.11. The standard deviation for all phantoms in all compression states cross the line at the ideal value $M=1$

With this experimental design, when reconstructing with UR the MRE phantom stiffness in the uncompressed and two compression states, it is observed that the material appears increasingly stiffer with increasing compression. This behaviour is observed in all seven phantoms, as shown in Fig. 8 (column 2). There it can be seen that, in the uncompressed case, the intrinsic stiffness is $\sim 4.5 \, \hbox {kPa}$. At the first compression level, the loading bias leads to an apparent stiffness of $\sim$ 6 kPa, and at the second compression level to an apparent stiffness of $\sim$ 7.5 kPa. A summary of the average stiffness of every phantom in each deformation state can be seen in Fig. 9. The statistics are based on a region of interest (as seen in Fig. 8) which excludes the phantom’s margins, in order to avoid peculiar boundary effects. A nonlinear stiffening trend can be observed with increased compression. The standard deviations increase as well, from $\sim$10 to $\sim$21%, suggesting a higher stiffness heterogeneity under compression, which could be a result of the complex wave behaviour. Since the phantom PVA material becomes apparently anisotropic under deformation, a shear wave (as in Eq. 13) probing, locally, different directions would lead to higher variability in the stiffness measurements.

Recall that in Sect. 2.1.3 we discussed the motion of a planar shear wave through a compressed body in order to gain an intuition of the biasing effects of loading. It was seen that, for an idealised planar wave (like in Eq. 15) moving through a compressed Neo-Hookean body, the material appears to be stiffer. Although the MRE experiment employed in this study carries additional complexity compared to the idealised case (particularly in the type of material used and wave structure), the stiffening trend observed in PVA is in accordance with the simplified theoretical outcomes just summarised. The same stiffening effect was observed in an ultrasound study as well (Gennisson et al. 2007), under similar conditions (idealised planar wave and uniaxially compressed material).

**Table 4:** MRE minimisation parameters fixing $\alpha =0.03$; the error, computed as per Eq. 23, can be seen in the first column, in brackets

Phantom	$C_2$ (Pa)	$\delta _1$ (Pa)	$\alpha$
p7 (15.85%)	93.68	2119.92	0.03
p8 (15.86%)	83.15	2085.28	0.03
p9 (14.80%)	88.36	1878.05	0.03
p10 (16.64%)	94.83	2126.26	0.03
p11 (17.08%)	103.43	2235.11	0.03
p12 (18.27%)	106.27	1945.07	0.03
p13 (11.15%)	246.54	1848.72	0.03
	Linear		Nonlin
Mean	120.43	2019.75	NA
SD	56.96	139.60	NA

### Intrinsic stiffness estimation

By understanding how loading biases stiffness estimation, it is possible to estimate intrinsic stiffness values when using CR. This requires knowledge of the deformation employed and the material law. Since this study deals with pure compression, the deformation was estimated using Eq. 12. Rheological testing of the PVA led to a 3-parameter material law (Eq. 24). It was seen, in Sect. 3.1, that there is variation in estimating the linear parameters $C_2$ and $\delta _1$, whereas the nonlinear parameter $\alpha$ is stable around 0.02–0.03. The optimisation performed in rheology was not repeated for MRE, as this was observed to lead to a parameter coupling between $\alpha$ and $\delta _1$. Thus, $\alpha$ was fixed at 0.03, as indicated by the rheological tests, and $C_2$, $\delta _1$ were estimated as described in the data analysis Sect. 2.3.2. The resulting parameters are presented in Table 4. It is notable that the $C_2$ parameter is generally lower in MRE (Table 4) than in rheology (Table 3), with the exception of phantom 13. This could be related to the degree of relative compression achieved. In rheology, the maximum relative compression is 28.5%. In MRE, however, relative compressions reach up to 35%, with the exception of phantom 13 (only 18%), as seen in Fig. 9. At lower deformation levels, the viscoelastic component is less employed; thus, the purely elastic part becomes more dominant. As $C_2$ is scaling the elastic component, its estimate is higher when less uniaxial compression is achieved. Within each MRE phantom, experimental imperfections lead to variability between pixels. Looking at the apparent stiffness mapsin Fig. 8, excluding the boundary area (as shown in column 2), a small degree of heterogeneity is observed, which accentuates under higher compressions. One reason behind this is speculated to lie in the crystallisation process during the F-T cycles of PVA. Assuming that the PVA freezes first at the mould edges and then in the centre, this could affect the local structures formed. Another reason could be that the top side of the phantoms is not perfectly flat, as it was cut manually. As such, the compression could have not been ideally uniform. Other arguments could revolve around the compression that the piston is exerting on the phantom, but an analysis in this sense is intricate and beyond the purpose of this study. Regardless of the underlying reasons, the small local variations in each phantom lead to the errors presented in Table 4, as slightly different pixels are attempted to be fit to the same measure. Although the errors are higher than in rheology (Table 3), the error metrics are not directly comparable. In rheology, a transient behaviour is investigated, that is, a data point is followed in time (Eq. 20), whereas in MRE a large spatial sample size is examined—all the pixels across several slices of each phantom, in uncompressed and second compressed state (Eq. 23).

The parameters obtained in Table 4 were sought by employing the CR on each phantom in uncompressed and second compression states, as previously described. Subsequently, they were used directly in reconstructing the corrected maps *M* for the first compression case, correspondingly for each phantom. The corrected maps *M* are illustrated, in a phantom, in Fig. 8 (column 3). Since the parameters are fit to best describe the data in uncompressed and second compressed states, it was indeed expected that the *M* map’s average for these cases is close to unity. Additionally, the corrected map for the first compression case, whose average also lies close to unity, demonstrates the prediction value of the parameters.

The quantified average of map *M* for all phantoms in all deformation states (uncompressed, first compression and second compression) is shown in Fig. 9 (right). Same as for the apparent stiffness $G^{\prime }$, only a ROI was considered inside each phantom, to avoid boundary effects. Depending on each phantom’s height, a variable compression $\lambda$ describes the first or second compression. For instance, the data points on the graph corresponding to $\lambda$ between 0.75 and 0.8 depict the first compression for taller phantoms and second compression for shorter phantoms. It is observed that the standard deviations are sizeable (10–19%) and tend to be higher at the larger compression levels. In the stiffness measurements, this observation was attributed to the apparent anisotropy effect and waves’ local probing directions. In the corrected maps *M*, the apparent anisotropy effect is undone by incorporating the knowledge on the deformation gradient, given by Eq. 12. Since the variance does not decrease in the *M* maps compared to $G^{\prime }$ maps, it suggests that the predicted apparent anisotropy in compression is not the main cause of the increasing standard deviations. Instead, it could be due to experimental imperfections which are difficult to control. For instance, the slight unevenness of the phantoms’ top or the compression induced by the piston indentation are unavoidable, yet for simplicity their effects are not accounted for in the deformation gradient, which employs an idealised form. Despite these, in all but one cases, the average of corrected map *M* lies within 10% of the ideal value of 1. This indicates that the model, parameterised with the values presented in Table 4, captures the bias induced by loading on the wave motion. This shows that the parameters found can describe the material at different deformation states and are suitable for estimating the intrinsic stiffness of the PVA material.

Integrating rheology and MRE is a difficult problem to tackle. While rheology can be employed to directly measure a material’s stress-strain response, MRE relies on investigating the wave propagation behaviour in order to estimate material properties. Here, we tried to integrate the two methods by validating a viscoelastic model using rheological data and then using it to derive the $\varvec{\mathcal {G}} ^{\prime }$ and $\varvec{\mathcal {G}} ^{\prime \prime }$ moduli to be used within CR of MRE data. The model was shown to capture the loading bias, thus proving the successful integration of the methods. Furthermore, model parameterisation was done separately for rheology and MRE, yet it is notable that the standard deviations around the mean of the $C_2$ and $\delta _1$ parameters are overlapping across the two experimental methods. This, together with the undoing of the loading bias, demonstrates the improvement that the newly developed CR brings in analysing MRE data.

### Extension and impact in tissue

The theoretical framework presented in Sect. 2.1.2 showed that the stiffness moduli are directly related to the material law and deformation. The work done in phantoms supports the theoretical concept. It was shown that, by incorporating the knowledge on material behaviour and deformation into the MRE reconstruction, the intrinsic stiffness can be estimated. This approach could be extended and applied in tissues. As a first step, a biomechanical model describing the tissue could be inferred and further adapted from animal rheological work. Additionally, by employing registration tools on anatomical images of an organ in undeformed and deformed states, deformations can be quantified. Together, a better characterisation of specific tissue rheology and large deformation recordings using MRI could enable proper interpretation of MRE data.

The most immediate extension of our approach could be breast MRE. This imaging technique relies on the breast being fixed in between two plates, which leads to tissue compression. In the literature, the loading bias was observed in cases where the breast was fixed tightly, compared to cases where the breast was fixed more loosely (Sinkus et al. 2005a), but was not accounted for. Undoing this bias using subject specific deformation would help in correcting the observed stiffness for both healthy and diseased areas. For instance, provided that the risk of breast cancer is associated with tissue stiffness (Boyd et al. 2014), accounting for the loading bias would ensure that the risk assessment is not underestimated or overestimated. Clinically, this could improve the prognostic value and disease management.

Another extension of our study could be in the liver, which is subjected to motion due to respiration. While this results mainly in translational motion, parts of the liver can be strained (Kang et al. 2012). This can be advantageous, since some liver pathologies were shown to become more apparent, compared to healthy tissue, under large strains (in ex vivo) (Yeh et al. 2002). Measuring stiffness in strained tissue could increase diagnostic accuracy, but would require techniques to eliminate loading bias.

The heart is a particularly complex organ which presents structural anisotropy, has dynamic stiffness, and undergoes large motion during the cardiac cycle. The latter constitutes one of the many challenges in assessing the unbiased stiffness of the myocardium (Kolipaka et al. 2010; Glaser et al. 2012), since the effects of deformation non-trivially interfere with the intrinsic tissue properties. The framework presented in this study would constitute a step forward in assessing the tissue characteristics, by eliminating the deformation bias.

In order to follow this paper’s proposed pathway of retrieving intrinsic material parameters from MRE data, one would have to consider Eq. 9 in the material frame as a starting point. Since the moduli $\varvec{\mathcal {G}} ^{\prime }$ and $\varvec{\mathcal {G}} ^{\prime \prime }$ depend on constitutive behaviour and deformation (e.g. Eq. 10), then knowledge on the PK2 tensor and deformation gradient is necessary. The material behaviour can be modelled by considering a constitutive equation for the strain energy function *W*, like in Eq. 17, from which tensor ${\varvec{S}}$ can be derived, or by proposing an adaption for ${\varvec{S}}$, e.g. Eq. 19. A formulation for the deformation gradient ${\varvec{F}}$ is required, like the one presented in Eq. 12, which needs to describe the unperturbed macro-deformation that the body is undergoing. From these, a form of the moduli $\varvec{\mathcal {G}} ^{\prime }$ and $\varvec{\mathcal {G}} ^{\prime \prime }$ can be inferred, which can be employed within CR (Eq. 22) to eliminate the loading bias.

### Study limitations

A limitation encountered during the analysis process of the data presented in this study was the estimation of the viscous modulus from MRE data. The rheological tests performed on PVA phantoms identified a very low $\alpha$, indicating that there is little viscous response of the material. This is a consequence of choosing a highly elastic material, with low wave attenuation. As such, the characteristics of the viscous component were difficult to identify under MRE testing, leading to an overestimation of the reconstructed viscous modulus. This effect was already identified with the employed MRE reconstruction (Fovargue et al. 2018a), where the overestimation of the viscous modulus was observed in a purely elastic phantom, particularly with increased noise. However, this drawback is predicted to disappear when using in vivo data, as tissue has a stronger viscous component. Nevertheless, in this phantom study, the bias due to the spectrum of stiffness prevented the characterisation of the full complex modulus, thus shifting the focus only on the real stiffness modulus.

This study provides the means through which a material’s intrinsic characteristics can be retrieved, despite it experiencing loading. However, a reasonable quantification of intrinsic material parameters relies on a good estimation of the deformation state and on an appropriate constitutive material law. In more complex scenarios, like in vivo measurements, these prerequisites are more challenging to attain. The deformation metrics would presumably need to rely on precise image registration techniques. As medical image registration is a greatly developed field, identification of suitable methods is surely attainable. However, the material constitutive law could be difficult to model, particularly in tissues which exhibit anisotropy. Nonetheless, much work has been done in this sense, as tissue models have been developed in many forms—polynomial, exponential, logarithmic, power laws, etc., and can be further improved and adapted. Undeniably, a model with increased complexity would lead to convoluted $\varvec{\mathcal {G}} ^{\prime }$ and $\varvec{\mathcal {G}} ^{\prime \prime }$ moduli, yet they can be attained numerically (by contrast with the analytic form presented here, for PVA, in Appendix 2). Hence, despite an increased computational cost, the use of a complex model would not be hindered.

## Conclusions

This paper presents a framework for integrating knowledge on material constitutive behaviour and deformation into wave dynamics in order to retrieve intrinsic material stiffness properties. First off, a theoretical foundation was established by perturbing Cauchy’s equations of motion and transcribing the resulting equations into material frame. This determined a form of the stiffness moduli which was seen to depend on the material model and deformation metrics. To support the theory, experiments on PVA phantoms were employed. The PVA material was tested in a rheological setup and its examined behaviour was modelled using a viscoelastic law. The same material was subjected to MRE experiments, where a known deformation (uniaxial compression) was employed. It was seen that, under the designed testing conditions, using standard MRE analysis, the material appeared to be stiffer with increasing compression. Thus, the known material model and deformation were integrated into the stiffness moduli and subsequently into the MRE reconstruction. By doing this, it was shown that intrinsic material stiffness parameters can be estimated, thus undoing the loading bias observed using standard analysis. Hence, this study provides a framework demonstrated to work in phantoms, which can be adapted and applied to MRE in more complex instances.

## Simplification to linearised elastic wave equations

Here we aim to simplify the total system of Eq. 8 by Perturbation theory, expanding about the current state ${\varvec{U}}$.

Starting by linearising the mass conservation equation, using the matrix determinant multiplication rule (Golub and Loan 1996), note that the first-order $\varepsilon$ terms of the Jacobian $J^\varepsilon$ may be clustered as^3^

25 $$\begin{aligned} J({\varvec{F}}^{\varepsilon })= & {} J+ D_{{\varvec{u}}}[J][{\varvec{u}^{\varepsilon }} ] \nonumber \\= & {} J + D_{{\varvec{u}}}[J({\varvec{F}})][{\varvec{u}^{\varepsilon }} ] \nonumber \\= & {} J + \frac{\partial J}{\partial {\varvec{F}}} : D_{{\varvec{u}}}[{\varvec{F}}][{\varvec{u}^{\varepsilon }} ] + {\mathcal {O}}(\varepsilon ^2) \nonumber \\= & {} J+ \frac{\partial J}{\partial {\varvec{F}}} : \nabla _0 {\varvec{u}^{\varepsilon }} + {\mathcal {O}}(\varepsilon ^2). \end{aligned}$$

Since $\frac{\partial J}{\partial {\varvec{F}}} = J {\varvec{F}}^{-T}$ (Bonet and Wood 2008), it follows that

26 $$\begin{aligned} J({\varvec{U}} + \varvec{u}^{\varepsilon })\approx & {} J + J {\varvec{F}}^{-T} : \nabla _0 \varvec{u}^{\varepsilon }. \end{aligned}$$

This approximation, considered to hold strongly when truncated to the first-order, reduces the mass balance in Eq. 8b to

27 $$\begin{aligned}&J^\varepsilon - 1 = J (1+ {\varvec{F}}^{-T} : \nabla _0 \varvec{u}^{\varepsilon })-1 = 0 \nonumber \\&\quad \text { on } {\varOmega }_0. \qquad \end{aligned}$$

Here, note that *J* is assumed to satisfy the original balance law in Eq. 3b, which leads Eq. 27 to become

28 $$\begin{aligned} {\varvec{F}}^{-T} : \nabla _0 \varvec{u}^{\varepsilon }= & {} 0. \end{aligned}$$

Using Eqs. 25 and 28, it is now clear that $J^\varepsilon = J + {\mathcal {O}}(\varepsilon ^2)$, which simplifies the momentum term in Eq. 8a to

29 $$\begin{aligned} \rho J^\varepsilon \partial _{tt} {\varvec{U}}^\varepsilon = \rho J \partial _{tt} {\varvec{U}}^\varepsilon + {\mathcal {O}}(\varepsilon ^2). \end{aligned}$$

To further reduce the momentum balance, we want to expand the stress term $\varvec{F}^\varepsilon {\varvec{S}}^\varepsilon$ in Eq. 8a using Taylor expansion in $(P, \varvec{F})$. As a result, letting ${\varvec{S}} = {\varvec{S}}_e(\varvec{C}(\varvec{F})) + D_t^{\alpha } {\varvec{S}}_v(\varvec{C}(\varvec{F})) + {\varvec{S}}_p ({\varvec{C}}({\varvec{F}}), P),$ using $\nabla _{\varvec{F}}$ to denote the derivative with respect to $\varvec{F}$, i.e.

$$\begin{aligned} (\nabla _{\varvec{F}})_{ij} = \partial / \partial F_{ij}, \end{aligned}$$

and the deltas for the difference between the perturbed and unperturbed states, i.e.

30 $$\begin{aligned} \begin{aligned} \delta \varvec{F}&= \varvec{F}^\varepsilon - \varvec{F}= \nabla _0 \varvec{u}^{\varepsilon }, \\ \delta P&= P^\varepsilon - P = p^{\varepsilon }, \end{aligned} \end{aligned}$$

we may focus on the deformation gradient expansion

31 $$\begin{aligned} \varvec{F}^\varepsilon= & {} \varvec{F}+ \sum _{n=1}^\infty \big ( [\nabla _{\varvec{F}}]^n {\varvec{F}} \big ) [: \delta \varvec{F}]^n , \nonumber \\\approx & {} \varvec{F}+ \nabla _0 \varvec{u}^{\varepsilon }+ {\mathcal {O}}(\varepsilon ^2) , \end{aligned}$$

the hydrostatic part expansion

32 $$\begin{aligned} {\varvec{S}}^\varepsilon _p= & {} {\varvec{S}}_p + \sum _{n=1}^\infty \Bigg ( \big ( [\nabla _{\varvec{F}}]^n {\varvec{S}}_p \big ) [: \delta \varvec{F}]^n \nonumber \\&+\,\bigg ( \frac{\partial ^n}{\partial P^n} {\varvec{S}}_p \bigg ) [: \delta P]^n \Bigg ) \nonumber \\\approx & {} \big ( {\varvec{S}}_p + \nabla _{\varvec{F}}{\varvec{S}}_p : \nabla _0 \varvec{u}^{\varepsilon }+ p^{\varepsilon }J \varvec{F}^{-T} \big ) + {\mathcal {O}}(\varepsilon ^2), \end{aligned}$$

the PK2 elastic part expansion

33 $$\begin{aligned} {\varvec{S}}^\varepsilon _e= & {} {\varvec{S}}_e + \sum _{n=1}^\infty \big ( [\nabla _{\varvec{F}}]^n {\varvec{S}}_e \big ) [: \delta \varvec{F}]^n , \nonumber \\\approx & {} {\varvec{S}}_e + \nabla _{\varvec{F}}{\varvec{S}}_e : \nabla _0 \varvec{u}^{\varepsilon }+ {\mathcal {O}}(\varepsilon ^2) , \end{aligned}$$

and the PK2 viscoelastic part expansion

34 $$\begin{aligned} D_t^\alpha {\varvec{S}}^\varepsilon _v= & {} D_t^\alpha \left( {\varvec{S}}_v + \sum _{n=1}^\infty \big ( [\nabla _{\varvec{F}}]^n {\varvec{S}}_v \big ) [: \delta \varvec{F}]^n \right) \nonumber \\\approx & {} D_t^{\alpha } \left( {\varvec{S}}_v + \nabla _{\varvec{F}}{\varvec{S}}_v : \nabla _0 \varvec{u}^{\varepsilon }\right) + {\mathcal {O}}(\varepsilon ^2) \nonumber \\\approx & {} \nabla _{\varvec{F}}{\varvec{S}}_v : D_t^{\alpha } \left( \nabla _0 \varvec{u}^{\varepsilon }\right) + {\mathcal {O}}(\varepsilon ^2), \end{aligned}$$

where it is assumed that ${\varvec{S}}_v$ and $\nabla _{\varvec{F}}{\varvec{S}}_v$ are constant in time.

Hence, the components of the stress term $\varvec{F}^\varepsilon {\varvec{S}}^\varepsilon$ are given by

35 $$\begin{aligned} \varvec{F}^\varepsilon {\varvec{S}}^\varepsilon\approx & {} \varvec{F}{\varvec{S}} \nonumber \\&+ \,\big ( \varvec{F}[\nabla _{\varvec{F}}({\varvec{S}}_e + {\varvec{S}}_p) ] + [\varvec{{\mathcal {I}}}] {\varvec{S}} \big ): \nabla _0 \varvec{u}^{\varepsilon }\nonumber \\&+ \,\varvec{F}[\nabla _{\varvec{F}}{\varvec{S}}_v] : D_t^{\alpha } \left( \nabla _0 \varvec{u}^{\varepsilon }\right) + p^{\varepsilon }J \varvec{F}^{-T} \nonumber \\&+ \, {\mathcal {O}}(\varepsilon ^2), \end{aligned}$$

where the square bracketed terms denote fourth-order tensors resulting from differentials of the PK2 stress tensor with respect to $\varvec{F}$. For clarity, in indicial notation,

36 $$\begin{aligned} (F^\varepsilon S^\varepsilon )_{ij}= & {} F_{ik}S_{kj} + F_{ik}\frac{\partial (S_e + S_p)_{kj} }{\partial F_{mn}} \frac{\partial u^\varepsilon _m}{\partial X_n} \nonumber \\&+ \,D_t^\alpha F_{ik} \frac{\partial (S_v)_{kj} }{\partial F_{mn}} \frac{\partial u^\varepsilon _m}{\partial X_n} + \frac{\partial u^\varepsilon _i}{\partial X_n} S_{nj} \nonumber \\&+ \,p^{\varepsilon }J F_{ji}^{-1} + {\mathcal {O}}(\varepsilon ^2). \end{aligned}$$

Thus, considering Eqs. 29 and 35, the conservation of momentum can be split between macro-deformation (in square brackets) and micro-deformation components, i.e.

37 $$\begin{aligned}&\Big [ \rho J \partial _{tt} {\varvec{U}}- \nabla _0 \cdot (\varvec{F}{\varvec{S}}) \Big ] + \rho J \partial _{tt} {\varvec{u}^{\varepsilon }} \nonumber \\&\quad -\, \nabla _0 \cdot \bigg ( \big [ \varvec{F}\nabla _{\varvec{F}}({\varvec{S}}_e + {\varvec{S}}_p) + \varvec{{\mathcal {I}}} {\varvec{S}} \big ]: \nabla _0 \varvec{u}^{\varepsilon }\nonumber \\&\quad +\, [\varvec{F}\nabla _{\varvec{F}}{\varvec{S}}_v] : D_t^{\alpha } \left( \nabla _0 \varvec{u}^{\varepsilon }\right) + p^{\varepsilon }J \varvec{F}^{-T} \bigg ) = \varvec{0}. \end{aligned}$$

Noting that the macro-deformation ${\varvec{U}}$ satisfies Eq. 3a, the first square bracketed term on the left hand side is zero. Hence, a set of linearised elastic wave equations in the reference frame can be formulated:

38a $$\begin{aligned}&\rho J \partial _{tt} {\varvec{u}^{\varepsilon }} - \nabla _0 \cdot \bigg ( \big [ \varvec{F}\nabla _{\varvec{F}}({\varvec{S}}_e + {\varvec{S}}_p) + \varvec{{\mathcal {I}}} {\varvec{S}} \big ]: \nabla _0 \varvec{u}^{\varepsilon }\nonumber \\&\quad + \,[\varvec{F}\nabla _{\varvec{F}}{\varvec{S}}_v] : D_t^{\alpha } \left( \nabla _0 \varvec{u}^{\varepsilon }\right) + p^{\varepsilon }J \varvec{F}^{-T} \bigg ) = \varvec{0}. \end{aligned}$$

38b $$\begin{aligned}&{\varvec{F}}^{-T} : \nabla _0 \varvec{u}^{\varepsilon }=0. \end{aligned}$$

While the set of Eq. 38 deals generically with small scale perturbations, in the case where the frequency is sufficiently high or the time interval sufficiently long, harmonic waves may be considered. Thus, inserting the wave form given by Eq. 2 into the set of Eq. 38, and noting the Laplace transform as $D_t^\alpha \left( e^{i \omega t} \right) \approx (i \omega )^\alpha e^{i \omega t} \, \,\forall t>>0,$ it follows that

39a $$\begin{aligned}&-\rho J \omega ^2 \varvec{u}_{{c}}-\nabla _0 \cdot \Big ( [{\varvec{F}} \nabla _{\varvec{F}}({\varvec{S}}_e + (i \omega )^\alpha {\varvec{S}}_v + {\varvec{S}}_p) \nonumber \\&\quad + \varvec{{\mathcal {I}}} {\varvec{S}} ] : \nabla _0 \varvec{u}_{{c}}+ p_{{c}}J {\varvec{F}}^{-T} \Big ) = \, \varvec{0} , \end{aligned}$$

39b $$\begin{aligned}&{\varvec{F}}^{-T} : \nabla _0 \varvec{u}_{{c}}= \, 0. \end{aligned}$$

When comparing with real data, it is desirable to map the momentum equations into the physical frame. Using the property (Ogden 1997) that

$$\begin{aligned} \nabla \widetilde{\varvec{y}} = (\nabla _0 \varvec{y}) \varvec{F}^{-1}, \end{aligned}$$

the gradients with respect to the reference frame can also be transformed into the current domain. Hence, the terms in the set of Eq. 39 become

40 $$\begin{aligned}&[{\varvec{F}} \nabla _{\varvec{F}}({\varvec{S}}_e + (i \omega )^\alpha {\varvec{S}}_v + {\varvec{S}}_p) + \varvec{{\mathcal {I}}} {\varvec{S}} ] : \nabla _0 \varvec{u}_{{c}}\nonumber \\&\quad = [{\varvec{F}} \nabla _{\varvec{F}}({\varvec{S}}_e + (i \omega )^\alpha {\varvec{S}}_v + {\varvec{S}}_p) + \varvec{{\mathcal {I}}} {\varvec{S}} ] : [ \nabla _0 \varvec{u}_{{c}}\varvec{F}^{-1} \varvec{F}] \nonumber \\&\quad = [\varvec{F}\nabla _{\varvec{F}}({\varvec{S}}_e + (i \omega )^\alpha {\varvec{S}}_v + {\varvec{S}}_p) \varvec{F}^T + \varvec{{\mathcal {I}}} {\varvec{S}} \varvec{F}^T ] : \nabla {\varvec{u}_{{c}}}, \nonumber \\ \end{aligned}$$

and

41 $$\begin{aligned} {\varvec{F}}^{-T} : \nabla _0 \varvec{u}_{{c}}= & {} {\varvec{F}}^{-T} : [\nabla _0 \varvec{u}_{{c}}\varvec{F}^{-1} \varvec{F}] \nonumber \\= & {} \varvec{{I}} : \nabla { \varvec{u}_{{c}}} \nonumber \\= & {} \nabla \cdot { \varvec{u}_{{c}}}. \end{aligned}$$

In indicial notation,

$$\begin{aligned}&\Big ( [{\varvec{F}} \nabla _{\varvec{F}}({\varvec{S}}_e + (i \omega )^\alpha {\varvec{S}}_v + {\varvec{S}}_p) + \varvec{{\mathcal {I}}} {\varvec{S}} ] : \nabla _0 \varvec{u}_{{c}}\Big )_{ij} \\&\quad = F_{ik} \frac{\partial (S_e+ (i \omega )^\alpha S_v + S_p)_{kj}}{\partial F_{mn}} \frac{\partial {{(u_c)}_m}}{\partial x_l} F_{ln} \\&\qquad +\, \delta _{im} S_{nj} \frac{\partial {{(u_c)}_m}}{\partial x_l} F_{ln}, \end{aligned}$$

and

$$\begin{aligned} {\varvec{F}}^{-T} : \nabla _0 \varvec{u}_{{c}}= & {} \frac{\partial {{(u_c)}_m}}{\partial x_m}. \end{aligned}$$

Moreover, by noticing the property linking the divergence of a tensor between the physical and reference domains (Gurtin et al. 2010)

$$\begin{aligned} J \nabla \cdot \widetilde{\varvec{H}}= & {} \nabla _0 \cdot [ J \varvec{H} \varvec{F}^{-T} ] \, , \phantom {\frac{a}{b}} \end{aligned}$$

the divergence of the stress terms can be recast in terms of the physical gradient operator $\nabla$, i.e.

42 $$\begin{aligned}&\nabla _0 \cdot \Big ( [ \varvec{F}\nabla _{\varvec{F}}({\varvec{S}}_e + (i \omega )^\alpha {\varvec{S}}_v + {\varvec{S}}_p) \varvec{F}^T \nonumber \\&\quad +\, \varvec{{\mathcal {I}}} {\varvec{S}} \varvec{F}^T ] : \nabla {\varvec{u}_{{c}}} \Big ) = J \nabla \cdot ( \varvec{\mathcal {C}} : \nabla {\varvec{u}_{{c}}} ) , \end{aligned}$$

where the fourth-order tensor $\varvec{\mathcal {C}}$ encompasses

43 $$\begin{aligned} {\mathcal {C}}_{ijml}= & {} \frac{1}{J} \bigg ( F_{ik} \frac{\partial (S_e + (i \omega )^\alpha S_v + S_p)_{ks}}{\partial F_{mn}} \nonumber \\&+\,\frac{\partial F_{ik}}{\partial F_{mn}} S_{ks} \bigg ) F_{ln} F_{js}. \end{aligned}$$

Combining Eqs. 40–42 and noting the property linking the divergence of a scalar in the reference domain to the physical frame (Gurtin et al. 2010)

$$\begin{aligned}&J \nabla \widetilde{w} = \nabla _0 \cdot [w J \varvec{F}^{-T}] \end{aligned}$$

the equations analogous to the classical set of Eq. 3, may be written as,

44a $$\begin{aligned}&-\rho J \omega ^2 \varvec{u}_{{c}}- J \nabla \cdot \Big (p_{{c}}\varvec{I} + \varvec{\mathcal {C}} : \nabla \varvec{u}_{{c}}\Big ) = \varvec{0} , \end{aligned}$$

44b $$\begin{aligned}&\nabla \cdot \varvec{u}_{{c}}= 0 , \nonumber \\&\quad \text {on } {\varOmega }. \end{aligned}$$

Equation 44a can be written as

45a $$\begin{aligned} -\rho \omega ^2 \varvec{u}_{{c}}- \nabla \cdot [ (\varvec{\mathcal {G}} ^\prime + i \varvec{\mathcal {G}} ^{\prime \prime } ): \nabla \varvec{u}_{{c}}+ p_{{c}}\varvec{I}]= & {} \varvec{0}, \end{aligned}$$

with the understanding that the real and imaginary scaling moduli $\varvec{\mathcal {G}} ^\prime$ and $\varvec{\mathcal {G}} ^{\prime \prime }$ are given by $\varvec{\mathcal {G}} ^{\prime } = \text {Re}( \varvec{\mathcal {C}} ), \, \varvec{\mathcal {G}} ^{\prime \prime } = \text {Im}( \varvec{\mathcal {C}} )$, i.e.

46a $$\begin{aligned} \varvec{\mathcal {G}} ^{\prime }= & {} \frac{1}{J} \Big ({\varvec{F}} \nabla _{\varvec{F}}{\varvec{S}}_e + \omega ^\alpha \cos \left( \frac{\pi \alpha }{2} \right) {\varvec{F}} \nabla _{\varvec{F}}{\varvec{S}}_v \nonumber \\&+\, {\varvec{F}} \nabla _{\varvec{F}}{\varvec{S}}_p + \varvec{{\mathcal {I}}} {\varvec{S}} \Big ) {\varvec{F}}^T {\varvec{F}}^T, \end{aligned}$$

46b $$\begin{aligned} \varvec{\mathcal {G}} ^{\prime \prime }= & {} \frac{\omega ^\alpha }{J} \sin \left( \frac{\pi \alpha }{2} \right) {\varvec{F}} \nabla _{\varvec{F}}{\varvec{S}}_v {\varvec{F}}^T {\varvec{F}}^T. \end{aligned}$$

## Viscoelastic moduli estimates for PVA material

In this section, the aim is to understand the effect of macrodeformations on the wave behaviour in a viscoelastic material described by Eq. 19. So far, general elastic and viscoelastic stresses ${\varvec{S}}_e$ and ${\varvec{S}}_v$ have been used. Recall that, for this work, they were considered to comprise two parts each, based on a Mooney–Rivlin type of law, as follows:

$$\begin{aligned}&{\varvec{S}}_e = C_1 {\varvec{S}}_{e1} + C_2 {\varvec{S}}_{e2}, \\&D_t^\alpha {\varvec{S}}_v = \delta _1 D_t^\alpha {\varvec{S}}_{e1} + \delta _2 D_t^\alpha {\varvec{S}}_{e2}. \end{aligned}$$

Hence, the fourth-order tensor $\varvec{\mathcal {C}}$ can be taken one step further and written as

48 $$\begin{aligned} {\mathcal {C}}_{ijml}= & {} \frac{1}{J} \Bigg \{ F_{ik}\Bigg [ {C_1} \frac{\partial S_{e1,ks}}{\partial F_{mn}} + {C_2} \frac{\partial S_{e2,ks}}{\partial F_{mn}} \nonumber \\&+\, (i \omega )^\alpha \Big ( {\delta _1} \frac{\partial S_{v1,ks}}{\partial F_{mn}} + {\delta _2} \frac{\partial S_{v2,ks}}{\partial F_{mn}} \Big )\nonumber \\&+\, \frac{\partial S_{p,ks}}{\partial F_{mn}} \Bigg ] + \frac{\partial F_{ik}}{\partial F_{mn}} S_{ks} \Bigg \} F_{ln} F_{js}, \end{aligned}$$

while the $\varvec{\mathcal {G}} ^{\prime }$ and $\varvec{\mathcal {G}} ^{\prime \prime }$ moduli become

49a $$\begin{aligned} \varvec{\mathcal {G}} ^{\prime }= & {} \frac{1}{J} \Bigg \{ {\varvec{F}} \bigg [ C_1 (\nabla _{\varvec{F}}{\varvec{S}}_{e1}) + C_2 (\nabla _{\varvec{F}}{\varvec{S}}_{e2}) \nonumber \\&+\, \nabla _{\varvec{F}}{\varvec{S}}_p \bigg ] + \varvec{{\mathcal {I}}} {\varvec{S}} \Bigg \} {\varvec{F}}^T {\varvec{F}}^T \nonumber \\&+\, \frac{\omega ^\alpha }{J} \cos \left( \frac{\pi \alpha }{2} \right) {\varvec{F}} \Bigg \{ \delta _1 (\nabla _{\varvec{F}}{\varvec{S}}_{e1}) \nonumber \\&+ \,\delta _2 (\nabla _{\varvec{F}}{\varvec{S}}_{e2}) \Bigg \} {\varvec{F}}^T {\varvec{F}}^T , \end{aligned}$$

49b $$\begin{aligned} \varvec{\mathcal {G}} ^{\prime \prime }= & {} \frac{\omega ^\alpha }{J} \sin \left( \frac{\pi \alpha }{2} \right) {\varvec{F}} \bigg \{ \delta _1 (\nabla _{\varvec{F}}{\varvec{S}}_{e1}) \nonumber \\&+ \,\delta _2 (\nabla _{\varvec{F}}{\varvec{S}}_{e2}) \bigg \} {\varvec{F}}^T {\varvec{F}}^T. \end{aligned}$$

The moduli $\varvec{\mathcal {G}} ^{\prime }$ and $\varvec{\mathcal {G}} ^{\prime \prime }$ are influencing the wave dynamics $\nabla \varvec{u}_{{c}}$, leading to an apparent wave dynamic. Now, considering the analytic formulation of the contraction $(\varvec{\mathcal {G}} ^\prime + i \varvec{\mathcal {G}} ^{\prime \prime } ): \nabla \varvec{u}_{{c}}$, consider that the wave $\nabla \varvec{u}_{{c}}$ satisfies the harmonic wave motion described in Sect. 2.1.1. After a cumbersome derivation and simplification process, the moduli defining the PVA material used can be formulated as

50 $$\begin{aligned}&\varvec{\mathcal {G}} ^{\prime } : \nabla \varvec{u}_{{c}}\nonumber \\&\quad =\frac{1}{J} \Bigg \{ C_1 \bigg [-\frac{4}{3} {\varvec{I}} (\widehat{{{\varvec{B}}}}:\nabla {\varvec{u}_{{c}}}) + \frac{2}{3} I_{\widehat{ {\varvec{C}}}} \nabla \varvec{u}_{{c}}^T + 2 \nabla \varvec{u}_{{c}}\widehat{{{\varvec{B}}}} \bigg ] \nonumber \\&\qquad +\, 8 C_2 \bigg [ (\widehat{{{\varvec{B}}}}^2 :\nabla { \varvec{u}_{{c}}}) \left( 4 \widehat{{{\varvec{B}}}}^2 + 4{\varvec{I}} - \frac{8}{3} II_{\widehat{{\varvec{C}}}} {\varvec{I}} \right) \nonumber \\&\qquad +\,\Big ( \widehat{{{\varvec{B}}}} \nabla \varvec{u}_{{c}}\widehat{{{\varvec{B}}}} + (\widehat{{{\varvec{B}}}} \nabla \varvec{u}_{{c}}\widehat{{{\varvec{B}}}})^T +\nabla \varvec{u}_{{c}}\widehat{{{\varvec{B}}}}^2 + \frac{II_{\widehat{{\varvec{C}}}}}{3} \nabla \varvec{u}_{{c}}^T \Big ) \nonumber \\&\qquad \cdot \,(II_{\widehat{{\varvec{C}}}} -3) \bigg ] - JP \nabla \varvec{u}_{{c}}^T \nonumber \\&\qquad +\, \omega ^{\alpha } \cos \left( {\frac{\pi \alpha }{2}} \right) \cdot \Bigg ( \delta _1 \frac{2}{3} I_{\widehat{{\varvec{C}}}} \big [ \nabla \varvec{u}_{{c}}+ \nabla \varvec{u}_{{c}}^T \big ] \nonumber \\&\qquad +\, 8 \delta _2 \bigg [ (\widehat{{{\varvec{B}}}}^2 :\nabla {\varvec{u}_{{c}}}) \left( 4 \widehat{{{\varvec{B}}}}^2 + 2 {\varvec{I}} - 2 II_{\widehat{{\varvec{C}}}} {\varvec{I}} \right) \nonumber \\&\qquad + \,\Big ( \widehat{{{\varvec{B}}}} \nabla \varvec{u}_{{c}}\widehat{{{\varvec{B}}}} + (\widehat{{{\varvec{B}}}} \nabla \varvec{u}_{{c}}\widehat{{{\varvec{B}}}})^T + \frac{II_{\widehat{{\varvec{C}}}}}{3} \big ( \nabla \varvec{u}_{{c}}+ \nabla \varvec{u}_{{c}}^T \big ) \Big ) \nonumber \\&\qquad \, \cdot \,(II_{\widehat{{\varvec{C}}}} -3) \bigg ] \Bigg ) \Bigg \} \end{aligned}$$

and

51 $$\begin{aligned}&\varvec{\mathcal {G}} ^{\prime \prime } : \nabla \varvec{u}_{{c}}\nonumber \\&\quad =\frac{1}{J} \Bigg \{ \omega ^{\alpha } \sin \left( {\frac{\pi \alpha }{2}} \right) \cdot \Bigg ( \delta _1 \frac{2}{3} I_{\widehat{{\varvec{C}}}} \big [\nabla \varvec{u}_{{c}}+ \nabla \varvec{u}_{{c}}^T \big ] \nonumber \\&\qquad +\, 8 \delta _2 \bigg [ (\widehat{{{\varvec{B}}}}^2 :\nabla {\varvec{u}_{{c}}}) \left( 4 \widehat{{{\varvec{B}}}}^2 + 2 {\varvec{I}} - 2 II_{\widehat{{\varvec{C}}}} {\varvec{I}} \right) \nonumber \\&\qquad +\, \Big ( \widehat{{{\varvec{B}}}} \nabla \varvec{u}_{{c}}\widehat{{{\varvec{B}}}} + (\widehat{{{\varvec{B}}}} \nabla \varvec{u}_{{c}}\widehat{{{\varvec{B}}}})^T + \frac{II_{\widehat{{\varvec{C}}}}}{3} \big ( \nabla \varvec{u}_{{c}}+ \nabla \varvec{u}_{{c}}^T \big ) \Big ) \nonumber \\&\qquad \cdot \,(II_{\widehat{{\varvec{C}}}} -3) \bigg ] \Bigg ) \Bigg \}. \end{aligned}$$

In the particular case when there exists no macro-deformation, i.e. ${\varvec{F}} = {\varvec{I}}$, it follows that the real scaling modulus retains only the terms

52 $$\begin{aligned} \varvec{\mathcal {G}} ^{\prime } : \nabla \varvec{u}_{{c}}= & {} \frac{1}{J} \Bigg \{ 2 C_1 \big ( \nabla \varvec{u}_{{c}}+ \nabla \varvec{u}_{{c}}^T \big ) - JP \nabla \varvec{u}_{{c}}^T \nonumber \\&+\,2 \delta _1 \omega ^{\alpha } \cos \left( {\frac{\pi \alpha }{2}} \right) \left( \nabla \varvec{u}_{{c}}+ \nabla \varvec{u}_{{c}}^T \right) \Bigg \} , \end{aligned}$$

while the imaginary modulus reduces to

53 $$\begin{aligned} \varvec{\mathcal {G}} ^{\prime \prime } : \nabla \varvec{u}_{{c}}= \frac{2 \delta _1 \omega ^{\alpha }}{J} \sin \left( {\frac{\pi \alpha }{2}} \right) \left( \nabla \varvec{u}_{{c}}+ \nabla \varvec{u}_{{c}}^T \right) . \end{aligned}$$

## Footnotes

1 Einstein summation over repetitive indices is assumed.

2 Only dimensionality *m* was used as this case is relevant to this work, particularly $m=3$.
